# Crosstalk between Mitochondrial and Sarcoplasmic Reticulum Ca^2+^ Cycling Modulates Cardiac Pacemaker Cell Automaticity

**DOI:** 10.1371/journal.pone.0037582

**Published:** 2012-05-29

**Authors:** Yael Yaniv, Harold A. Spurgeon, Alexey E. Lyashkov, Dongmei Yang, Bruce D. Ziman, Victor A. Maltsev, Edward G. Lakatta

**Affiliations:** Laboratory of Cardiovascular Science, Intramural Research Program, National Institute on Aging, National Institutes of Health, Baltimore, Maryland, United States of America; Harvard Medical School, United States of America

## Abstract

**Background:**

Mitochondria dynamically buffer cytosolic Ca^2+^ in cardiac ventricular cells and this affects the Ca^2+^ load of the sarcoplasmic reticulum (SR). In sinoatrial-node cells (SANC) the SR generates periodic local, subsarcolemmal Ca^2+^ releases (LCRs) that depend upon the SR load and are involved in SANC automaticity: LCRs activate an inward Na^+^-Ca^2+^ exchange current to accelerate the diastolic depolarization, prompting the ensemble of surface membrane ion channels to generate the next action potential (AP).

**Objective:**

To determine if mitochondrial Ca^2+^ (Ca^2+^_m_), cytosolic Ca^2+^ (Ca^2+^_c_)-SR-Ca^2+^ crosstalk occurs in single rabbit SANC, and how this may relate to SANC normal automaticity.

**Results:**

Inhibition of mitochondrial Ca^2+^ influx into (Ru360) or Ca^2+^ efflux from (CGP-37157) decreased [Ca^2+^]_m_ to 80±8% control or increased [Ca^2+^]_m_ to 119±7% control, respectively. Concurrent with inhibition of mitochondrial Ca^2+^ influx or efflux, the SR Ca^2+^ load, and LCR size, duration, amplitude and period (imaged via confocal linescan) significantly increased or decreased, respectively. Changes in total ensemble LCR Ca^2+^ signal were highly correlated with the change in the SR Ca^2+^ load (r^2^ = 0.97). Changes in the spontaneous AP cycle length (Ru360, 111±1% control; CGP-37157, 89±2% control) in response to changes in [Ca^2+^]_m_ were predicted by concurrent changes in LCR period (r^2^ = 0.84).

**Conclusion:**

A change in SANC Ca^2+^_m_ flux translates into a change in the AP firing rate by effecting changes in Ca^2+^_c_ and SR Ca^2+^ loading, which affects the characteristics of spontaneous SR Ca^2+^ release.

## Introduction

Mitochondrial Ca^2+^ flux plays a fundamental role buffering cytosolic Ca^2+^ (Ca^2+^_c_) in cardiac ventricular myocytes (VM) in normal and pathological conditions [1]. Ca^2+^_c_ enters mitochondria through the mitochondrial uniporter and is extruded by the mitochondrial Na^+^-Ca^2+^ exchanger [2]. Mitochondrial Ca^2+^ buffering modulates both cell and sarcoplasmic reticulum (SR) Ca^2+^ load [1], [3], and on this basis affects VM contractility. In response to β-adrenergic stimulation, shifts in VM mitochondrial Ca^2+^ flux mediate an increase in energy supply required for increased contractility.

Sinoatrial nodal cells (SANC) initiate each heart beat by generating spontaneous APs that emanate from the sinoatrial node. Numerous studies over the last decade indicate a crucial role for SR cycling in SANC normal automaticity, giving rise to a coupled-clock system which explains the basis of robust regulation of SANC function [4], [5], [6] : Intracellular Ca^2+^ cycling comprises a “Ca^2+^ clock” within the coupled-clock system and the ensemble of surface membrane electrogenic molecules comprises the system’s membrane clock. Unlike VM, even in the absence of β-adrenergic stimulation in SANC, high levels of basal cAMP and cAMP-mediated protein kinase A-dependent (PKA) and Ca^2+^/calmodulin-dependent protein kinase II phosphorylation of Ca^2+^ cycling-proteins of both clocks enables the SR (Ca^2+^ clock) to generate spontaneous rhythmic local, subsarcolemmal Ca^2+^ releases (LCRs) via SR ryanodine receptors. LCRs activate an inward Na^+^-Ca^2+^ exchange current that accelerates the rate of diastolic depolarization, prompting the membrane clock to generate the next action potential (AP).

We hypothesized that changes in Ca^2+^ flux into and out of mitochondria in SANC will directly affect SR Ca^2+^ loading, and thus indirectly affect SR Ca^2+^ release, and therefore that a change in mitochondrial Ca^2+^ (Ca^2+^_m_) cycling will modulate the normal automaticity of the coupled-clock system.

Changing the external electrical stimulation rate in VM is a convenient approach to induce changes in cross-talk between mitochondrial and SR Ca^2+^ cycling. But, this method can not be explored in SANC, which fire spontaneous AP’s. Therefore, to test our hypothesis that mitochondrial-SR Ca^2+^ crosstalk exists and links to SANC AP firing, we measured [Ca^2+^]_c_, [Ca^2+^]_m_, SR Ca^2+^ load and Ca^2+^ releases, and the spontaneous AP firing rate in intact, single, isolated SANC in response to specific inhibitors of Ca^2+^ flux into (Ru360) and Ca^2+^ flux from (CGP-37157) mitochondria.

## Results

### Selective Quenching of Cytosolic Indo-1 by Mn^2+^ Permits Assessment of [Ca^2+^]_m_ in SANC

**Figure 1:**
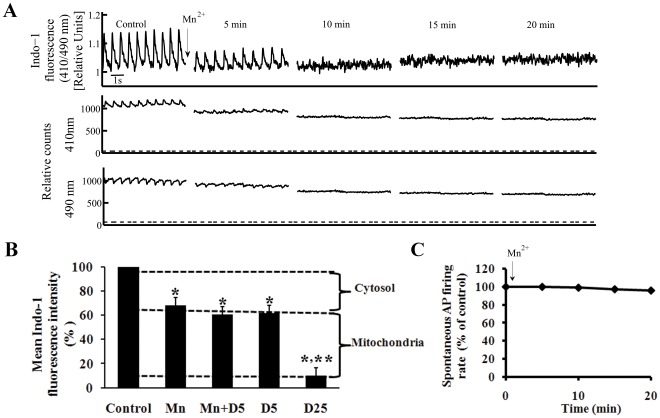
Validation of mitochondrial Ca^2+^ measurements. (A) Mn^2+^ (50 µmol/L) quenching of the cytosolic Indo-1 fluorescence ratio 410/490 (top panel), and fluorescence emission at wavelengths of 410 (middle panel), and 490 nm (lower panel). Dashed line under recordings of flourecence emission at individual wavelengths indicates background cell autofluorescence (the fluorescence without Indo-1 loading). (B) Permeabiliziation of the sarcolemma by 5 µmol/L digitonin alone (D5; n = 6) or together with Mn^2+^ to quench cytosolic Indo-1 (n = 6). Note that Mn^2+^ plus digitonin decreases the Indo fluorescence to the same level as Mn^2+^ alone (n = 10). A higher digitonin concentration (25 µmol/L D25; n = 4), permeabilizes the mitochondrial membrane in addition to the sarcolemma, and markedly depletes the cell fluorescence. (C) Application of Mn^2+^ does not alter the spontaneous AP firing rate. *p<0.05 vs. drug control, **p<0.05 vs. Mn^2+^.

Ca^2+^_m_ was indexed by selective Mn^2+^ quenching of the cytosolic fluorescence of the Ca^2+^ probe, Indo-1 [7]. Application of 50 µM MnCl_2_ to Indo-1-AM loaded SANC selectively quenched the cytosolic Indo-1 fluorescence ratio 410/490 in a time dependent manner (11±2 min), as evidenced by the disappearance of the AP-induced cytosolic Ca^2+^ transient (Fig. 1A). The change in Indo-fluorescence intensity at each wavelength is illustrated in lower panels in Fig. 1A. Note that following 15-min of exposure to Mn^2+^, the remaining fluorescence was stable. Importantly, 20 min of exposure to Mn^2+^ caused only a small, non-significant decrease in the spontaneous AP firing rate compared to control (Fig. 1C). Since our prior studies [4], [5], [6] have demonstrated a tight relationship between Ca^2+^_c_ and normal automaticity the lack of an appreciable effect of Mn^2+^ on SANC AP firing rate suggests that at this concentration Mn^2+^ has minimal effects on [Ca^2+^]_c_ or SR Ca^2+^ cycling.

To define the compartmental Indo-quenching, we quenched Indo-1 fluorescence using varying concentrations of digitonin: a low concentration of digitonin (5 µM) or saponin (25 µg/ml), which permeabilizes only the sarcolemma (Fig. S1 A-B); and a higher concentration of digitonin (25 µM), which also permeabilizes mitochondrial membranes (Fig. S1 C-D) [8], [9]. Figure 1B shows that Mn^2+^ treatment decreased the fluorescence to 68±2% of its control value. A low concentration of digitonin alone, or together with Mn^2+^ to quench cytosolic Indo-1, decreased the fluorescence to 63±4 and 61±3% control, respectively, i.e., to the same level as Mn^2+^ alone (p = 0.1 and 0.7, respectively). Saponin (25 µg/ml) reduced the fluorescence to 64±5% (n = 5), i.e. to the same level as permeabilizing of sarcolemma with the lower concentration of digitonin. Thus, when the SANC plasma membrane was selectively permeablized, the Indo fluorescence decreased to the same level as it did in cells with an intact surface membrane in the presence of Mn^2+^. In contrast, a higher concentration of digitonin, which also permeabilizes mitochondrial membranes, decreased the fluorescence to 10±4% control (p = 0.001, compared to the Mn^2+^ treated group). These results provide evidence that the Mn^2+^-resistant fluorescence of Indo-1 loaded cells is derived from Indo-1 within the mitochondrial compartment. Note, that in the time-control experiment protocol without Mn^2+^ application, Indo-1 fluorescence was only slightly quenched (−7±3%).

**Figure 2:**
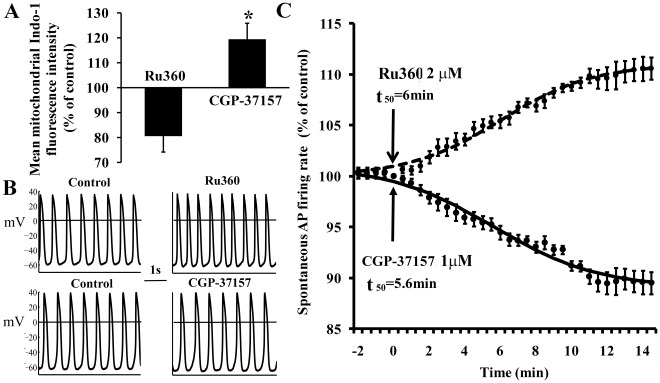
The effect of inhibition of mitochondrial fluxes on mitochondrial Ca^2+^ and spontaneous AP firing rate. (A) Mean mitochondrial fluorescence intensity in response to specific inhibition of mitochondrial Ca^2+^ influx or efflux (n = 10 for each drug), (B) AP recordings (before and after 15 min exposure to each of the drugs), and (C) average time-dependent change in the rate of AP induced contractions in the presence of CGP-37157 (n = 12) or Ru360 (n = 11). **p<0.05 vs. Ru360.

Ru360 and CGP-37157, specific inhibitors of Ca influx into and efflux from mitochondria, respectively, were used to perturb [Ca^2+^]_m_. Figure 2A shows that Ru360 (2 µM) decreased mean [Ca^2+^]_m_ to 80±8% of control (from 210±19 to 171±19 nM) of the Mn^2+^ quenched control; conversely, CGP-37157 (1 µM) increased [Ca^2+^]_m_ to 119±7% of control (from 210±19 to 270±13 nM) (p<0.05 Ru360 vs. CGP-37157, see Fig. S2 for representative examples). Thus, Ru360 and CGP-37157 affect Ca^2+^_m_ comparably in SANC as in previous studies in ventricular myocytes [10], [11].

### Perturbations of Ca^2+^_m_ Cycling Affect the SANC Spontaneous AP Firing Rate

The main question of our study is whether a change in Ca^2+^ cycling into and out of mitochondria can modulate normal automaticity. Figure 2B shows representative examples of the effect of a 15-min exposure of SANC to specific inhibitors of Ca^2+^ influx into or efflux from mitochondria on spontaneous SANC AP firing. The average time-courses of the change in AP firing rate in response to perturbations of mitochondrial Ca^2+^ efflux or influx are illustrated in Fig. 2C. Inhibition of Ca^2+^ influx into mitochondria by Ru360 (2 µM) caused, over a 10-min period, a progressive increase in the spontaneous SANC AP firing rate to 111±1% control (from 2.7±0.25 to 3±0.3 Hz). Conversely, inhibition of Ca^2+^ efflux from mitochondria by CGP-37157 (1 µM) progressively reduced the spontaneous AP firing rate to 89±2% control (from 2.65±0.2 to 2.3±0.2 Hz) over a 10-min period. Note that both effects evolved and saturated with roughly the same time course. These drug effects were not reversible even after 10-min wash. The changes in other AP parameters are summarized in Table 1.

**Table 1:** Average characteristics of SANC spontaneous APs in response to pharmacological perturbations (n = 9 in each group).

	Control	Ru360	Control	CGP-37157
**Frequency (Hz)**	2.7±0.25	3±0.3*	2.65±0.2	2.3±0.2* ^,^ **
**Action potential amplitude (mV)**	90±2	91±2	91±4	93±5
**Overshoot (mV)**	31±1	31±2	33±3	35±3
**Maximal diastolic potential (mV)**	−59±1	−60±1	−61±2	−62±2
**Max rate of diastolic** **depolarization, dV/dt (mV/s)**	6.2±1	6.8±1	6±1	5.8±1

* p<0.05 vs. drug control.

** p<0.05 vs. Ru360.

### Perturbations of SANC Ca^2+^_m_ Cycling Affect the SANC Ca^2+^_c_ Characteristics

To determine how Ca^2+^ buffering by the mitochondria affects [Ca^2+^]_c_ we loaded cells with Indo-1AM. Changes in spontaneous AP firing rate by inhibition of mitochondrial Ca^2+^ influx or efflux were determined in Indo-1/AM loaded cells (see representative examples in Fig. S2). Table 2 lists the average characteristics of the AP-induced Ca^2+^_c_ transients measured by Indo-1 and Table 3 list the average characteristics of the AP-induced Ca^2+^_c_ transients in the presence of Ru360 or CGP-37157. Inhibition of Ca^2+^_m_ influx significantly increased peak systolic Ca^2+^ from 380±23 to 415±28 nM (p = 0.001) and decreased the 90% decay time of cytosolic [Ca^2+^] (T-90_c_) from 302±13 to 285±11 ms (p = 0.04). The diastolic Indo fluorescence (p = 0.6), time to peak Ca^2+^ (T-P_c_; p = 0.07), and 50% decay time of cytosolic Ca^2+^ (T-50_c_; p = 0.2) were not significantly altered. Conversely, inhibition of Ca^2+^_m_ efflux significantly decreased peak systolic [Ca^2+^] from 369±13 to 342±13 nM (p = 0.02) and increased T-90_c_ from 301±14 to 325±12 ms (p = 0.04). The diastolic Indo fluorescence (p = 0.12), T-P_c_ (p = 0.3), and T-50_c_ (p = 0.2) were not significantly altered. Therefore, the effects of inhibition of [Ca^2+^]_m_ influx or efflux on [Ca^2+^]_c_ in SANC are similar to their effects in prior studies in guinea pig ventricular cardiac myocytes [12].

**Table 2:** Average characteristics of SANC spontaneous AP induced Ca^2+^ transients (n = 43).

**Peak systolic Ca^2+^ (nmol/L)**	374±8
**Minimum diastolic Ca^2+^ fluorescence (nmol/L)**	130±4
**Ca^2+^ transient amplitude** **(systolic-diastolic) (nmol/L)**	244±8
**T-P_c_ (ms)**	87±3
**T-50_c_ (ms)**	182±5
**T-90_c_ (ms)**	301±8
**Frequency (Hz)**	2.3±0.1

### Perturbation of [Ca^2+^]_m_ Cycling Affects SR Ca^2+^ Load

Numerous studies of SANC indicate that the characteristics of SR Ca^2+^ cycling are determined, in part, by the SR Ca^2+^ load. Perturbations of mitochondrial Ca^2+^ flux might affect SR Ca^2+^ loading if these two organelles were located substantially close to each other. To determine the proximity of mitochondria to SR we employed dual immunolabeling to SANC with anti-SERCA2 antibody and Mitotracker orange to visualize the SR and mitochondrial membrane, respectively. SANC of varying size exhibit robust uniform SERCA2 immunolabeling (Fig. S3 left panels) and robust mitochondrial labeling (Fig. S3 middle panels). The merged images (Fig. S3 right panels) indicate that a substantial part of the mitochondrial mass is colocalized with that of SERCA2. This colocalization would highly facilitate rapid mitochondrial-SR Ca^2+^ cycling.

**Figure 3:**
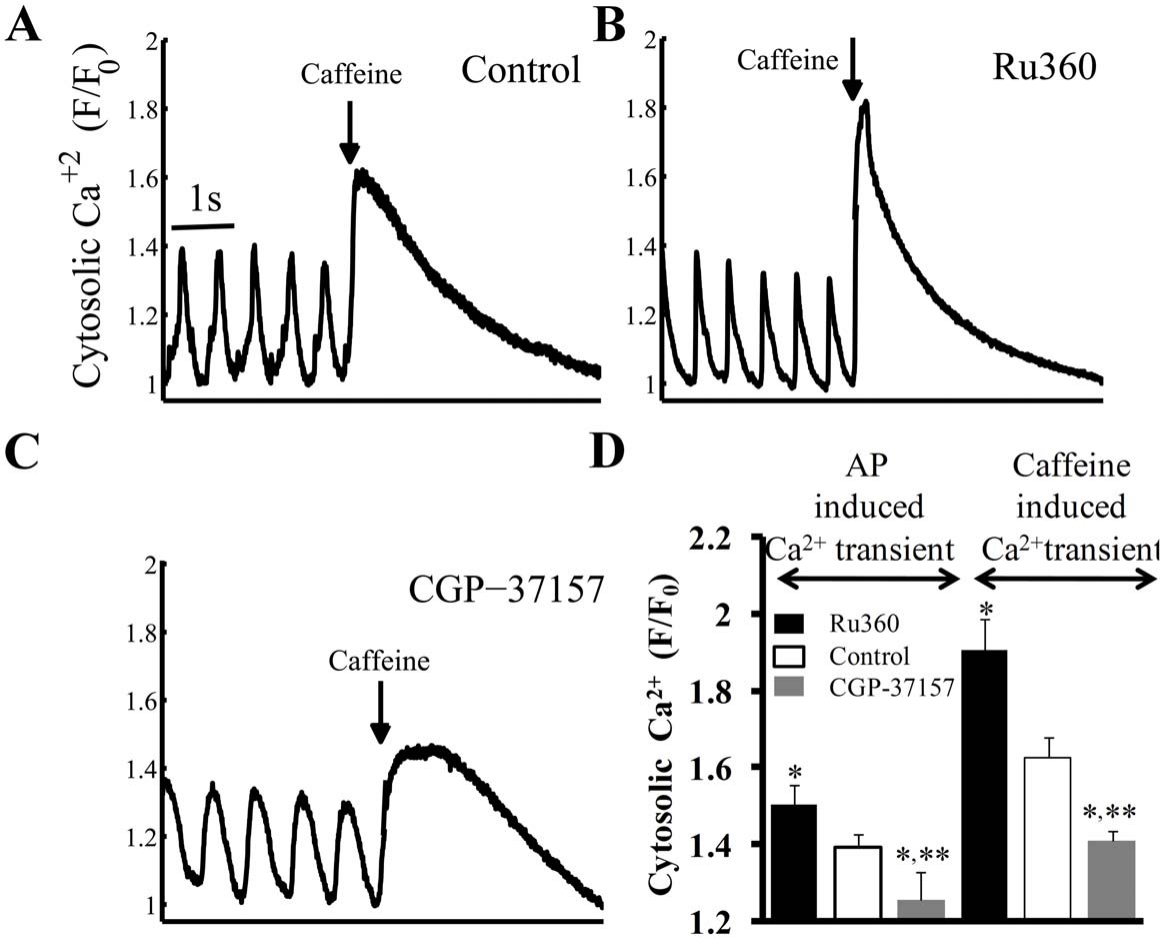
SR load estimation from rapid caffeine application. Effects of a rapid application (“spritz”) of caffeine (indicated by the arrow) onto SANC (A) in control, or (B) in the presence of Ru360 or (C) CGP-37157. (D) Average effects of Ru360 or CGP-37157 on peak AP-induced cytosolic Ca^2+^ prior to a caffeine spritz (left), and the subsequent caffeine-induced cytosolic Ca^2+^ transient (right) (n = 12, for each group). (The caffeine response can be usually measured only once in a given SANC, because following caffeine application a prolonged period is required for AP firing rate to return to the control AP firing rate. Therefore, the effects of caffeine before (i.e. control) and following application of drugs that affect Ca^2+^_m_ flux were measured in different cells).

Changes in AP triggered Ca^2+^ transient in response to perturbing Ca^2+^_m_ flux (Fig. 2C) could change the SR Ca^2+^ loading. Specifically, inhibition of Ca^2+^_m_ influx, while reducing [Ca^2+^]_m_ (Fig. 2A), might result in more Ca^2+^ within the cytosol available for pumping into the SR; conversely, inhibition of Ca^2+^_m_ efflux to increase [Ca^2+^]_m_ might “steal” Ca^2+^ from the cytosol, resulting in reduced Ca^2+^ pumping into the SR. To estimate changes in the SR content, brief rapid applications of caffeine were applied (“spritzed”) onto the cell following drug application to perturb Ca^2+^_m_ flux. Representative examples and average data are presented in Figures 3A–C and Fig. 3D (right bars), respectively. Blocking Ca^2+^ influx into mitochondria increased the SR Ca^2+^ load (assessed by change in amplitude of the Ca^2+^ transient induced by a brief rapid application of caffeine) by 17±5% of control (1.6±0.05 to 1.9±0.08 F/F_0_; p = 0.01); conversely inhibition of Ca^2+^ efflux from mitochondria reduced the SR Ca^2+^ load by 13±2% of control (1.6±0.05 to 1.4±0.03 F/F_0_; p = 0.03) (Fig. 3D, right bars). Although CGP-37157 reduced the rate of decay of the caffeine-evoked response compared to Ru360, this decrease was not significant. The change in the AP-induced Ca^2+^ transient amplitude by Ru360 or CGP-37157 prior to caffeine application (Fig. 3D, left bars) is similar to the trend measured by Indo-1 (Table 3). Note that the relative drug-induced changes in the caffeine and AP-induced cytosolic Ca^2+^ transient amplitudes and changes in Ca^2+^_m_ and cycle length are all roughly equivalent. Thus, the changes in the amplitude of systolic Ca^2+^_c_ transient due to manipulation of Ca^2+^_m_ flux, could, in part at least, be due to changes in the SR Ca^2+^ load. Although, Ru360 and CGP-37157 tended to increase and decrease, respectively, the PLB phosphorylation at Serine-16 (PKA-dependent site), these effects were not statistically significant (Fig. S4). Note, however, that isoproterenol, employed as a positive control, markedly increases PLB phosphorylation.

**Table 3:** Average characteristics of SANC spontaneous AP induced Ca^2+^ transients in response to pharmacological perturbations.

% control	Ru360 (n = 10; 2 µM)	CGP-37157 (n = 10; 1 µM)	CPA (n = 8; 5 µM)
**Peak Systolic Ca^2+^**	10±1* (p = 0.001)	−10±3* (p = 0.03)	−14±3* (p = 0.001)
**Diastolic Ca^2+^ fluorescence**	2±2 (p = 0.59)	−6±4 (p = 0.12)	−1±4 (p = 0.4)
**Ca^2+^ transient amplitude** **(systolic-diastolic)**	13±3* (p = 0.001)	−10±5* (p = 0.001)	−28±7* (p = 0.003)
**T-P_c_**	−6±5 (p = 0.2)	2±4 (p = 0.3)	10±5 (p = 0.14)
**T-50_c_**	−4±3 (p = 0.08)	3±3 (p = 0.2)	8±5* (p = 0.02)
**T-90_c_**	−6±2* (p = 0.03)	7±3* (p = 0.04)	19±7* (p = 0.04)
**Frequency**	10±2* (p = 0.004)	−10±3* (p = 0.02)	−33±4* (p<0.001)
**Mean mitochondrial** **fluorescence intensity**	80±8* (p = 0.02)	119±7* (p = 0.03)	86±8* (p = 0.045)

* p<0.05 vs. drug control.

### Perturbation of [Ca^2+^]_m_ Cycling Affects Spatiotemporal Characteristics of Rhythmic, Spontaneous SR-generated Local Ca^2+^ Releases (LCRs) via Ryanodine Receptors

**Figure 4:**
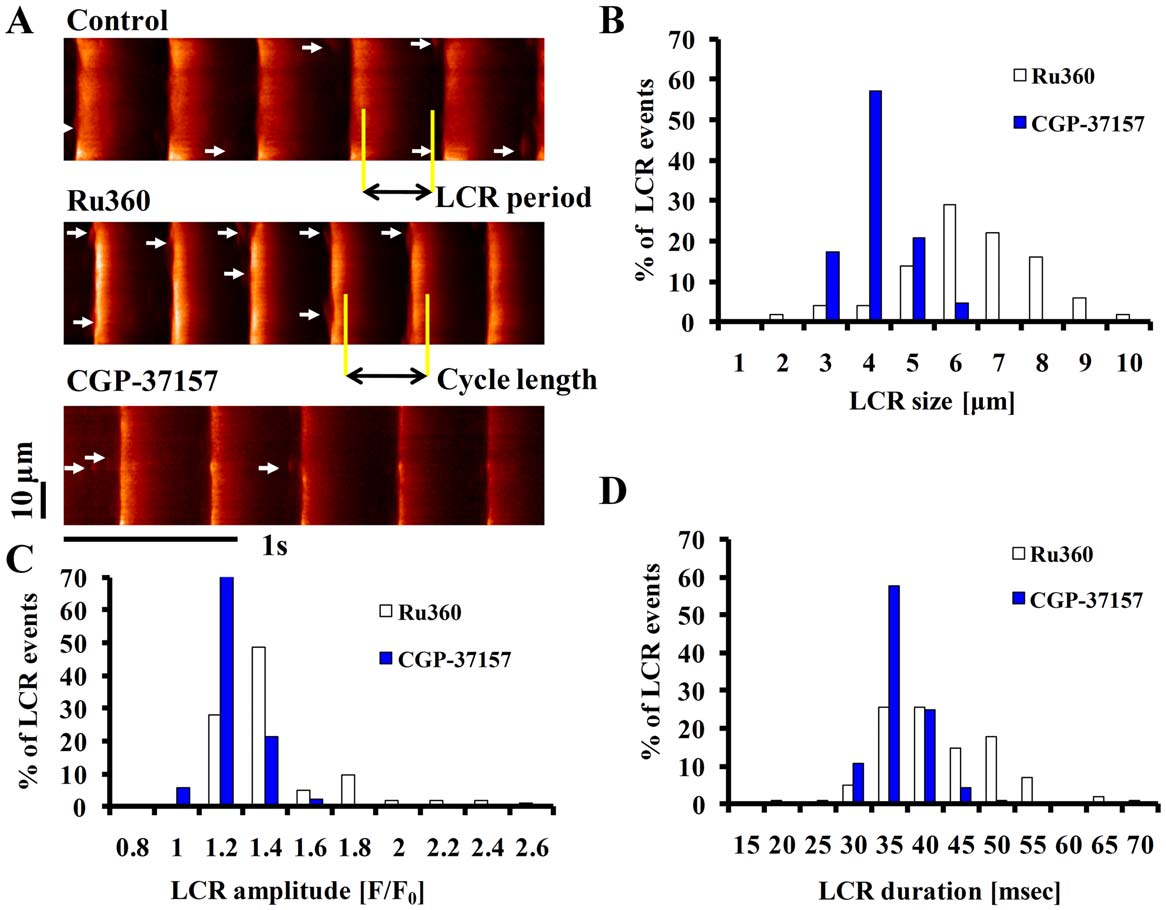
Specific inhibition of Ca influx into or efflux from mitochondria in intact SANC modifies spatiotemporal characteristics of LCRs. (A) Confocal line scan Ca^2+^ images of a representative SANC before and following exposure to 2 µmol/L Ru360 or 1 µmol/L CGP-37157. LCRs are indicated by arrowheads. The LCR period is defined as the time from the peak of the prior AP-induced Ca^2+^ transient to the LCR onset. Histograms of LCR (B) size (full width at half-maximum amplitude), (C) amplitude (F/F_0_), and (D) duration (full duration at half-maximum amplitude) in the presence of Ru360 (n = 12; 102 LCRs) and CGP-37157 (n = 12; 92 LCRs).

Prior studies have shown that changes in SR Ca^2+^ loading in SANC affect spontaneous diastolic LCRs [5], [13]. To determine how a perturbation of Ca^2+^_m_ cycling affects spatiotemporal characteristics of LCRs, we measured LCRs in Fluo-4 AM loaded SANC, and imaged its Ca^2+^-dependent fluorescence by confocal microscopy. Representative examples (Figure 4A) show that the spontaneous AP firing rate, measured via confocal line scan imaging increased (by Ru360) or decreased (by CGP-37157). Figures 4B-4D depict histograms of LCR size, amplitude, and duration in the presence of Ru360 or CGP-37157. Ru360 shifted distribution of LCR amplitudes, spatial widths, and durations to larger values. On average, Ru360 increased LCR size from 4.2±0.1 to 6.1±0.2 µm (p<0.001), the LCR amplitude from 1.24±0.02 to 1.36±0.04F/F_o_ (p = 0.04), and the LCR duration from 35±0.8 to 41±0.8ms (p<0.001). In contrast to Ru360, CGP-37157 decreased the average LCR size from 4.3±0.1 to 2.6±0.1 µm (p<0.001), the average LCR amplitude from 1.22±0.02 to 1.14±0.01F/F_o_ (p = 0.02), and the average LCR duration from 35±0.5 to 29±0.6ms (p<0.001).

**Figure 5:**
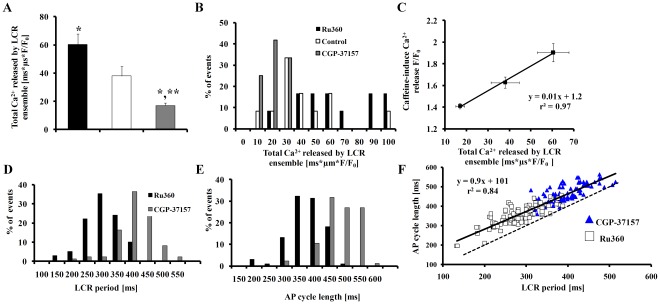
Relationships among the Ca^2+^ release of the LCR ensemble, LCR period, SR Ca^2+^ load and AP firing rate. The total LCR Ca^2+^ ensemble (A) average data and (B) histogram in the presence of Ru360 or CGP-37157. (C) The relationship between caffeine-induced Ca^2+^ release F/F_0_ and the total LCR Ca^2+^ ensemble. Specific inhibition of Ca^2+^ influx into or efflux from mitochondria in intact SANC shifts the LCR period and AP cycle length. (D) LCR period and (E) AP cycle length in the presence of Ru360 (n = 12; 102 LCRs) or CGP-37157 (n = 12; 92 LCRs). (F) The change in LCR period in response to perturbing mitochondrial Ca^2+^ flux predicts the concomitant change in AP cycle length. The Ru360-induced decrease in the spontaneous cycle length, and CGP-37157-induced increase in spontaneous cycle length, are both predicted by their effects on the LCR period. The dashed line is the line of identity. *p<0.05 vs. control, **p<0.05 vs. Ru360.

The total Ca^2+^ of the LCR ensemble shifted to lower values in response to CGP-37157, and to higher values in response to Ru360 (Fig. 5A–B). Figure 5A shows that, interestingly, Ru360 increased the ensemble LCR Ca^2+^ from 38±6 to 61±7 ms*µm*F/F_0_ (p<0.001) and CGP-37157 decreased it from 35±3 to 17±2 ms*µm*F/F_0_ (p = 0.003). The Ru360-induced increase, and the CGP-37157-induced decrease in the total LCR ensemble Ca^2+^ paralleled the effects of Ru360 and CGP-37157 on the amplitude of caffeine induced Ca^2+^ release into the cytosol (Fig. 5C). This suggests that changes in spontaneous LCRs elicited by Ru360 or CGP-37157 are linked to concomitant changes in SR Ca^2+^ load effected by these drugs.

**Figure 6:**
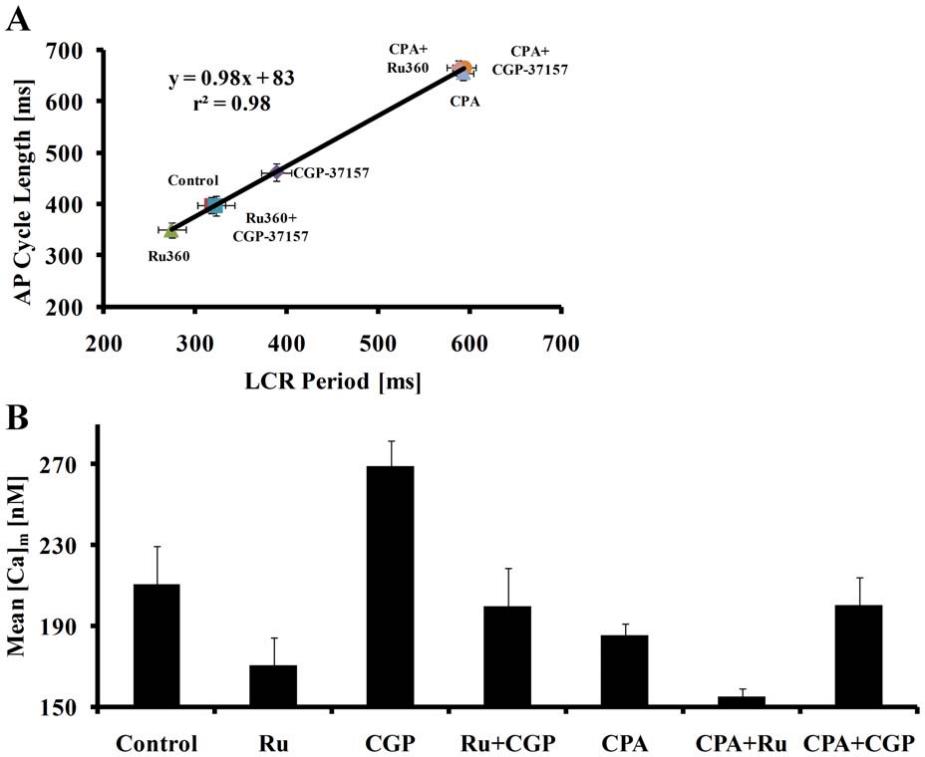
The effect of CPA on AP cycle length, LCR period and mitochondrial Ca^2+^. (A) The effect of CPA on the change in LCR period predicts the concurrent prolongation of AP cycle length. Note that this effect of CPA on LCR period and AP cycle length form a continuum with the effects of Ru360 and CGP-37157 on LCR period and AP cycle length. (B) In the presence of CPA neither Ru360 nor CGP-37157 affect the LCR period or AP cycle length, and [Ca^2+^]_m_ decreased. The combination of Ru360 plus CGP-37157 does not change [Ca^2+^]_m_, AP cycle length or the LCR period.

The LCR period, defined as the time from the peak Ca^2+^ transient (the onset SR Ca^2+^ release triggered by the prior AP) to an LCR onset (as illustrated in Fig. 4A) was reduced by Ru360 from 317±5 to 274±6ms (Fig. 5D, p<0.001), and increased by CGP-37157 to 389±7ms (Fig. 5D, p<0.001). Changes in the spontaneous AP cycle length occurred concomitantly with changes in the LCR period by specific inhibition of Ca^2+^ influx into or efflux from the mitochondria (Fig. 5E); change in LCR period (from 274±6 (Ru360) to 389±7ms (CGP-37157)) predicted the concomitant change in the spontaneous SANC AP cycle length from 349±5 to 462±6ms (Fig. 5F, r^2^ = 0.84, p<0.001). The relationship between the change in LCR period and AP cycle length by perturbations of Ca^2+^_m_ flux is quantitatively strikingly similar to that which occurs in response to other interventions which affect the LCR period and AP cycle length of SANC [14]. Interestingly, simultaneous application of Ru360 and CGP-37157 neither significantly altered Ca_m_, the spontaneous AP firing rate, nor LCR characteristics (Fig. 6). Thus, when both drugs are present the decrease in Ca^2+^ influx into the mitochondria by Ru360 can be compensated for by the decrease in Ca^2+^ efflux from the mitochondria by CGP-37157. Note, that this result only indicates that blocking both Ca^2+^ influx and efflux does not change the mitochondrial buffering capacity, and does not indicate that under physiological conditions mitochondrial fluxes do not play a role in modulating SR Ca^2+^ loading.

### Inhibition of SR Ca^2+^ Cycling Abolishes the Effect of Mitochondrial Ca^2+^ Cycling on the SANC Spontaneous AP Firing Rate

To prove that a change in SR load is a necessary component in the feedback mechanism between [Ca^2+^]_m_ and the AP firing rate, we reduced the SR load by blocking SR Ca^2+^ pumping with Cyclopiazonic acid (CPA) (5 µM) [15]. Both the LCR period (from 347±8 to 565±24 ms; p<0.001) and AP cycle length (from 413±7 to 594±20 ms; p<0.001) were markedly prolonged by CPA (Fig. 6A) and Ca^2+^_m_ decreased (Table 3). Note that changes in the spontaneous AP cycle length induced by CPA alone or by inhibition of mitochondrial Ca^2+^ flux, and changes in the LCR period form a continuum (Fig. 6A). Note also in Figure 6A, that in the presence of CPA the effects of Ru360 or CGP-37157 on the AP cycle length are eliminated. Moreover, the difference in [Ca^2+^]_m_ between Ru360 and CGP-37157 (Fig. 6B) is reduced in the presence of CPA, and therefore the effects of these drugs on mitochondrial Ca^2+^ buffering are reduced (Fig. 6B). To further test the idea that an increase in mitochondrial Ca^2+^ buffering decreases the SR Ca^2+^ load, we employed a sufficient concentration of CPA to reduce SR Ca^2+^ load to a similar extent as CGP-37157, and compared its effect on AP cycle length to those of CGP-37157. CPA (0.5 µM) decreased the spontaneous AP firing rate by 15±3% (n = 12, from 2.4±0.1 to 2±0.07 Hz; p = 0.003) and reduced the SR Ca^2+^ load by 17±3% (n = 12 in each group; from 1.5±0.09 to 1.2±0.05 F/F_0_; p = 0.003). This decrease in SR load by CPA is comparable to decreases in both spontaneous AP firing rate and SR Ca^2+^ load effected by CGP-37157. These results are consistent with our interpretation that the changes in AP cycle length due to manipulation of [Ca^2+^]_m_ content are linked to changes in the SR Ca^2+^ load.

### Blocking the “funny” Current, If, does not Affect the Mitochondrial Ca^2+^ Cycling Effect on the SANC Spontaneous AP Firing Rate

It had previously been thought that I_f_ is the main regulator of SANC spontaneous AP firing rate [16]. To determine the extent to which I_f_ effectively influences the effect of perturbing Ca^2+^_m_ cycling on the SANC spontaneous AP firing rate, we employed CsCl to inhibit I_f_. Figure S5 shows that application of CsCl plus Ru360 increased the beating rate, over a 15-min period, to 111±1% control (from 2.5±0.1 to 2.74±0.1 Hz; p = 0.003). Application of CsCl plus CGP-37157 decreased the beating rate, over a 15-min period, to 88±1% control (from 2.5±0.1 to 2.26±0.1 Hz; p = 0.01). These effects of I_f_ inhibition plus inhibition of mitochondrial Ca^2+^ fluxes neither quantitatively nor qualitatively differed from those when inhibitors of mitochondrial Ca^2+^ flux alone were applied without I_f_ inhibition. Therefore I_f_ does not have a role in the effects of Ru360 or CGP-37157 on AP firing rate (compare Fig. S5 with Fig. 2).

### Novel Numerical Model Simulations Predict the Changes in SANC Membrane Ionic Currents, SR and Mitochondrial Ca^2+^ Fluxes, and AP Cycle Length in Response to Ru360 or CGP-37157

To quantitatively simulate SR and mitochondrial dynamics and the AP firing rate when Ca^2+^_m_ is perturbed, and to simulate how surface membrane currents change when SR and mitochondrial Ca^2+^ fluxes are perturbed, we extended the SANC coupled-clock numerical model [17] to include mitochondrial Ca^2+^ fluxes. For details of the model and simulations see in Text S1 (the model parameters are in Table S1 and Table S2).

**Figure 7:**
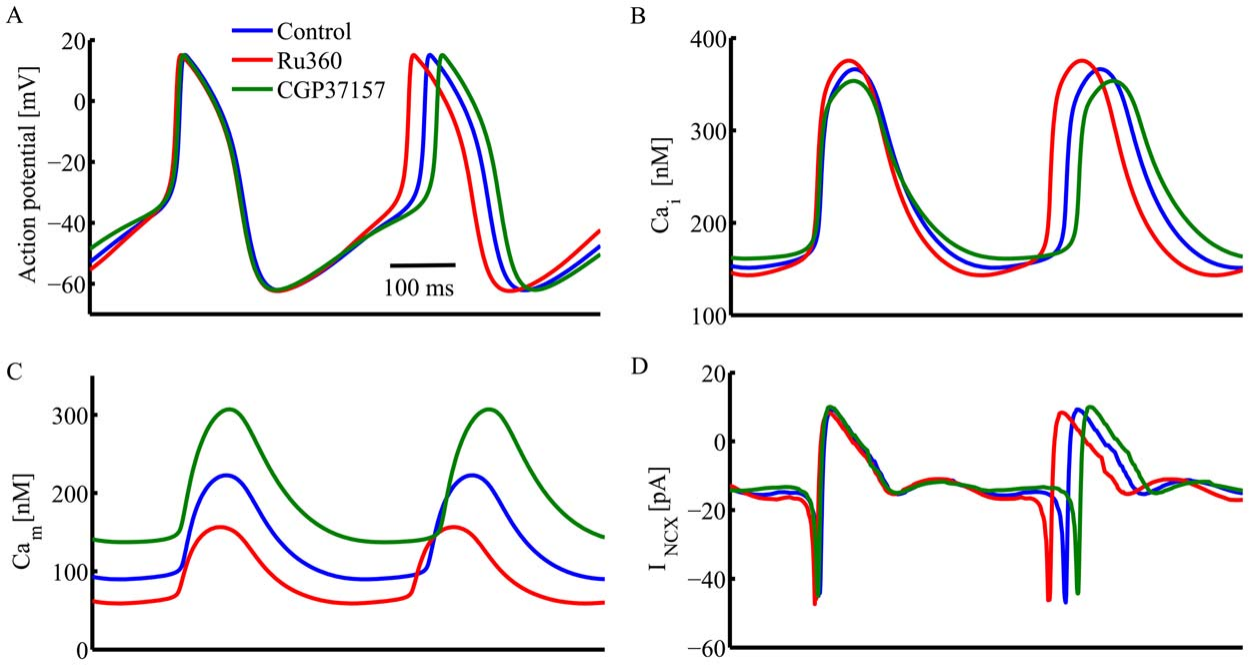
Simulations of the coupled clock numerical model, extended to include mitochondrial Ca^2+^ fluxes. The simulated effects of specific inhibition of Ca influx into or efflux from mitochondria on (A) membrane potential, (B) cytosolic Ca^2+^, (C) mitochondrial Ca^2+^, and (D) sarolemmal Na^+^-Ca^2+^ exchanger current in intact SANC.

The extended coupled clock numerical simulations reproduce the effect of manipulation of Ca^2+^_m_ on spontaneous AP firing rate (Fig. 7A) and Ca^2+^_c_ (Fig. 7B). The model simulations also predict that manipulation of mitochondrial Ca^2+^ flux perturbs the junctional and network SR Ca^2+^ fluxes, as observed experimentally (Fig. S6). This finding is in accord with our experimental observations that manipulation of mitochondrial Ca^2+^ flux affects the SR Ca^2+^ content. While our experimental data permit quantification of the average cytosolic and diastolic [Ca^2+^]_m_ only (see above), the extended coupled clock numerical simulations also predict both systolic and diastolic levels of [Ca^2+^]_m_, under basal conditions, and when mitochondrial Ca^2+^ flux is inhibited (Fig. 7C). Inhibition of mitochondrial Ca^2+^ influx by Ru360 decreased the maximum diastolic [Ca^2+^]_m_ (from 89.8 to 60 nM) and the systolic [Ca^2+^]_m_ (from 222.7 to 160 nM). Inhibition of mitochondrial Ca^2+^ efflux by CGP-37157, however, increased both diastolic [Ca^2+^]_m_ (to 137.2 nM) and systolic [Ca^2+^]_m_ (to 308 nM). Note, that manipulation of Ca^2+^_m_ affected the cycle length without affecting I_f_ kinetics, which is also consistent with our experimental result. Moreover, the model predicts that sarcolemmal Na^+^-Ca^2+^ exchanger current kinetics are altered indirectly due to change in LCR characteristics (Fig. 7D) when Ca^2+^_m_ flux is perturbed.

**Figure 8:**
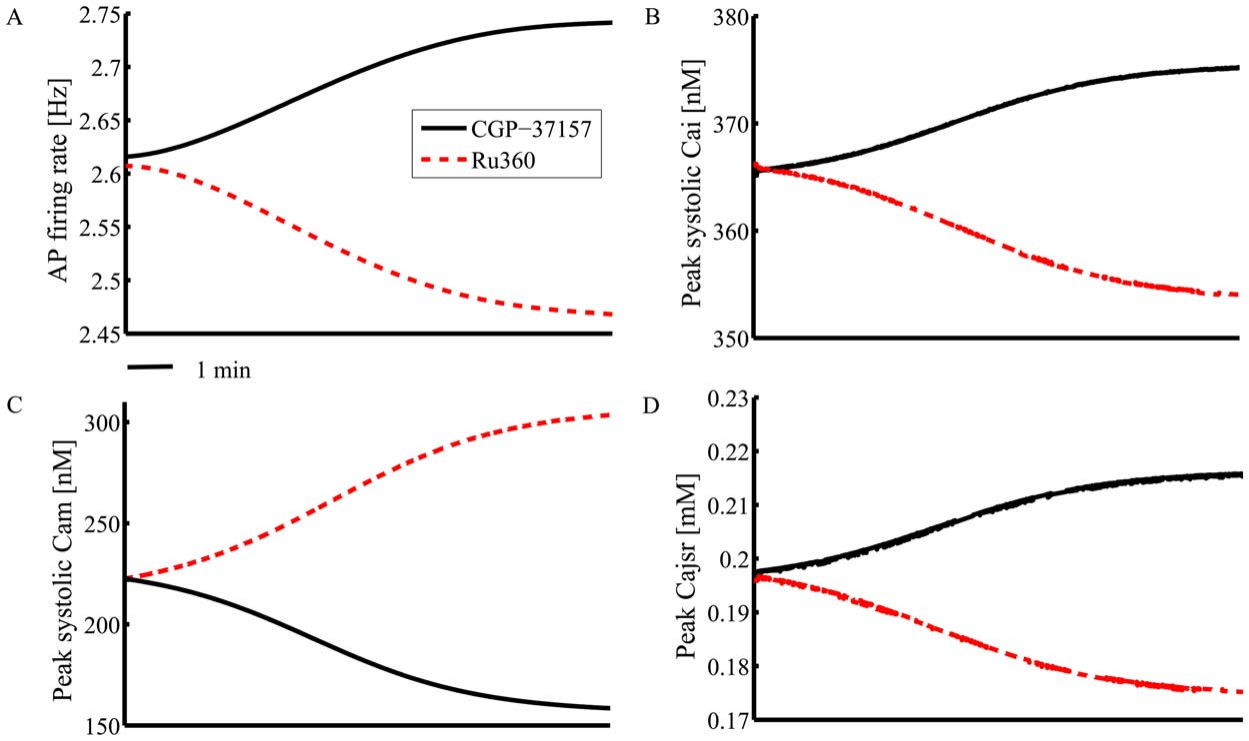
The extended coupled clock numerical model simulations of kinetics. The simulation of the change in (A) AP firing rate, (B) peak systolic cytosolic Ca^2+^, (C) peak systolic mitochondrial Ca^2+^, and (D) peak Ca^2+^ in junctional SR in response to specific inhibition of Ca^2+^ flux into or flux from mitochondria.

Since it is not possible experimentally to implement long recordings in isolated, single SANC (∼15 min) to measure the kinetics of the changes in intracellular Ca^2+^ dynamics and AP firing we used a numerical model to predict the inhibition of Ca^2+^ influx or efflux affects on cytosolic Ca^2+^, SR Ca^2+^ load, and AP firing rate. Model simulations predict that inhibition of mitochondrial Ca^2+^ efflux by CGP-37157 increases peak [Ca^2+^]_m_ over a 10-min time course and concomitantly decreases peak cytosolic Ca^2+^, peak jSR Ca^2+^ and spontaneous AP firing rate (Fig. 8). Simulation of inhibition of mitochondrial Ca^2+^ influx by Ru360 yielded a mirror image of the effect of CGP-37157. Thus these numerical simulations support the idea that when mitochondrial Ca^2+^ flux is perturbed, the changes in mitochondrial Ca^2+^, cytosolic Ca^2+^, SR Ca^2+^ loading and AP firing rate occur with the same kinetics.

In summary, we conclude that the coupled clock system of intracellular Ca^2+^ cycling proteins and surface membrane electrogenic molecules that regulates SANC normal automaticity includes SR-mitochondrial Ca^2+^ cross-talk.

## Discussion

The present results show, for the first time, that mitochondrial-SR Ca^2+^ cross talk, previously demonstrated in ventricular myocytes [18], also occurs within SANC. A most important novel finding of our study is that changes in Ca^2+^ cycling into and out of mitochondria in SANC, effected by using Ru360 and CGP-37157, modulate basal coupled-clock system automaticity via an impact on Ca^2+^_c_, SR loading and indirectly on Ca^2+^ release characteristics: blocking mitochondrial Ca^2+^ efflux increases [Ca^2+^]_m_, decreases the SR load, decreases the amplitude of the AP triggered cytosolic Ca^2+^ transient and total Ca^2+^ released spontaneously by the LCR ensemble, and reduces the AP firing rate. Conversely, reducing mitochondrial Ca^2+^ influx decreases [Ca^2+^]_m_, increases SR Ca^2+^ loading, increases the amplitude of the AP triggered cytosolic Ca^2+^ transient and total Ca^2+^ released spontaneously by the LCR ensemble, and increases the AP firing spontaneous rate (Fig. 3, 4, 5, Table 3). That interfering with mitochondrial Ca^2+^ flux modulates the spontaneous AP firing rate via effects on Ca^2+^_c_ and SR Ca^2+^ loading and release supports the idea that, in the absence of these mitochondrial Ca^2+^ flux inhibitors, mitochondrial buffering of Ca^2+^_c_ modulates SR Ca^2+^ cycling and AP firing. Since basal Ca^2+^-cAMP-PKA signaling within SANC also regulates ATP supply-demand matching, Ca^2+^_m_-Ca^2+^_c_ cross-talk indirectly links to ATP supply and demand mechanisms.

Mitochondrial-SR Ca^2+^ cross-talk observed in our experiments in SANC also occurs in other cell types. Observation in non-excitable cells indicate that the pattern and frequency of spontaneous cytosolic Ca^2+^ oscillations changes in the presence of CGP-37157, and it was argued, therefore, that mitochondrial Ca^2+^ flux may modulate cytosolic Ca^2+^ oscillations in these cells [19]. Also, the amplitude of spontaneous Ca^2+^ waves in Cajal cells originating from the mitochondrial area are attenuated by Ru360, while CGP-37157 suppressed the initial Ca^2+^ rise that reduced Ca^2+^ wave frequency [20]. In VM, differences in the kinetics of mitochondrial Ca^2+^ influx and efflux have been noted among species [12], [18], [21]. For example, Maack et al, similar to the present study, observed changes of [Ca^2+^]_c_ and [Ca^2+^]_m_ in guinea-pig cardiomyocytes when mitochondrial Ca^2+^ fluxes were perturbed by Ru360 or CGP-37157 [12]. In rat cardiomyocytes under basal conditions, in contrast to guinea-pig, application of Ru360 decreases Ca^2+^_m_, but does not alter Ca^2+^_c_ [11], [22]. Also of note, in isolated perfused guinea pig hearts, CGP-37157 has only a non-significant trend to reduce heart rate [3], whereas in the present study, the AP firing rate is significantly reduced by CGP-37157. Differences between the prior study and our study likely not only reflect species differences, but also the different experimental preparations employed: i.e., isolated single pacemaker cells in the present study vs. intact hearts in the prior study.

Whether mitochondrial Ca^2+^ buffers cytosolic Ca^2+^ on a beat-to-beat base in VM is a controversial issue related to uncertainty with respect to the extent and kinetics of both uptake and release of Ca^2+^ into and out the mitochondria [23], [24] (for a thoughtful review see [25]). To estimate the mitochondrial influx rate in VM, Ca^2+^_m_ was measured in response to different pacing rates (or during stimulation compared to quiescent mode). External stimulation in SANC is not feasible, however, because SANC beat spontaneously, and it is challenging to over-drive the spontaneous SANC AP firing rate, while maintaining a normal rhythm (Lakatta et al. unpublished observation). Beat-to-beat Ca^2+^_m_ flux into or out of the mitochondria of SANC in the present study can be roughly estimated, however assuming that Ru360 or CGP-37157 reduces the influx or efflux by 30%, respectively (based on our numerical model), and assuming a linear time-course of drug effects to inhibit their targets. Given the experimentally determined time course of the drug-induced changes in [Ca^2+^]_m_, and the average number of spontaneous AP cycles over this time, a rough estimation of drug-induced changes in Ca^2+^ flux from or into the mitochondria per AP cycle is on the order of 0.5 nM/beat.

The validity of our interpretations of the present results assumes that the method used to measure Ca^2+^_m_ does not substantially affect [Ca^2+^]_m_ itself, or affect other functions within SANC that are related to the AP firing rate. Because our major goal was to explore whether perturbations of mitochondrial Ca^2+^ cycling modulates the basal AP firing rate of SANC, neither cell permeabilization [26] which abolishes AP firing, nor dialysis of rhod-2 [12] is suitable to achieve this goal (Dialysis is a method to measure [Ca^2+^]_m_ that requires acute rupture of a membrane patch, often creating a small leak current, that notably affects the balance of ionic currents during diastolic depolarization and spontaneous AP frequency of SANC [27]). We selected the Mn^2+^ quench approach to assess [Ca^2+^]_m_ in SANC because measurements of [Ca^2+^]_m_ in VM by cell permeabilization and Mn^2+^ quench are in close agreement [26]. The ability of Mn^2+^ to selectively quench Ca^2+^_c_ fluorescence, without causing marked changes in Ca^2+^_m_, is based on its slower quenching of the mitochondrial fluorescence [7], [28]. Important requirements of this method are: 1) that the lowest concentration of Mn^2+^ that rapidly quenches cytosolic Ca^2+^but has minimal effects on sarcolemmal or mitochondrial Ca^2+^ ion transport be employed and 2) that during Ca^2+^_c_ quenching the temperature be maintained at a near physiological level. We employed 50 µM Mn^2+^ to quench Ca^2+^_c_ fluorescence, which at this concentration has no effect on our major functional endpoint, the spontaneous AP firing rate. Specifically, since we previously have shown [4], [5], [6] a tight relationship between cell cycling Ca^2+^ and AP firing rate, the lack of a sustained effect of Mn^2+^ on the SANC spontaneous AP firing rate strongly suggests that there is no functionally significant inhibition of sarcolemmal Ca^2+^ channels (i.e. L-type channels) by 50 µM Mn^2+^. Another concern about the Mn^2+^ quench technique is that Mn^2+^ can inhibit oxidative phosphorylation. The lack of a sustained effect of Mn^2+^ on the basal spontaneous AP firing rate again suggests that Mn^2+^ does not perturb the matching of ATP supply to demand, since we have previously shown that this matching is closely linked to the AP firing rate [29].

The selectivity of drugs employed to perturb Ca^2+^_m_ flux is another important issue that merits consideration in interpreting the present results. We employed Ru360 at concentration of 2 µM to selectively block Ca^2+^ influx into the mitochondria, i.e. a concentration similar to that used in prior studies in VM [11], [12] to effectively block Ca^2+^ influx to the mitochondria and to decrease [Ca^2+^]_m_. In rat VM, in which Ca^2+^_c_ is not substantially affected by changes in Ca^2+^_m_, this concentration of Ru360 does not affect SR Ca^2+^ uptake or L-type current [11]. Moreover, under basal conditions in rat VM Ru360 neither affects mitochondrial membrane potential nor ROS production [30]. Further, CPG-37157 does not likely increase the probability of mPTP opening, which generates ROS, because even during cardiac glycoside toxicity, application of CGP-37157 together with ouabain reduces oxidative species production that is effected by ouabain itself, even though Ca^2+^_m_ is higher during ouabain application than during control conditions [3].

We employed CGP-37157 at concentration of 1 µM because it selectively blocks the mitochondrial Na^+^-Ca^2+^ exchanger (IC_50_ = 0.5 µM) [31] and increases [Ca^2+^]_m_, but has only minor effects on sarolemmal Na^+^-Ca^2+^ exchanger (IC_50_ = 13 µM), or Ca^2+^ uptake by SR (IC_50_ = 10 µM) or on ryanodine receptor open probability (IC_50_ = 9.5 µM) [32]. L-type Ca^2+^ current is a crucial element of SANC automaticity [14]. Although CGP-37157 has been reported to affect L-type current in atrial cells (IC_50_ = 0.3 µM) [33], in rat even 10 µM Ru360 VM does not affect L-type current [11]. Our experimental evidence (Fig. 6) indicates that simultaneous application of Ru360 and CGP-37157 in SANC cancel each other’s effect on automaticity, suggesting that CGP-37157 does not have a substantial effect on L-type current in SANC. Moreover, the lack of effect of Ru360 or CGP-37157 on SANC AP parameter characteristics (Table 1) strongly suggests that there is no significant functional inhibition of any sarcolemmal channels by either of these drugs applied as in our study.

Our novel numerical model simulations reproduce our experimental findings and validate our interpretation of these findings: changes in mitochondrial Ca^2+^ flux affect the spontaneous AP firing rate of SANC via changes in SR Ca^2+^ loading and release. Furthermore, while our experimental method to measure [Ca^2+^]_m_ does not permit evaluation of systolic and diastolic [Ca^2+^]_m_, our model predicts that under basal conditions both diastolic and systolic [Ca^2+^]_m_ are lower than the diastolic and systolic [Ca^2+^]_c_, respectively. However, the systolic [Ca^2+^]_m_ is higher than diastolic [Ca^2+^]_c_.

### Summary

In summary, the present study shows, for the first time, that SANC mitochondria and SR are closely associated, i.e., that Ca^2+^ cycling into and out of the mitochondria acts as a dynamic buffer of cytosolic Ca^2+^, thus affecting Ca^2+^ availability for SR Ca^2+^ loading and release. When mitochondrial Ca^2+^ influx or Ca^2+^ efflux is selectively blocked, SR Ca^2+^ cycling and Ca^2+^ load are altered. This leads to changes in spontaneous AP triggered SR Ca^2+^ release that produce changes in both global AP induced cytosolic Ca^2+^ transients and to spontaneous, localized submembrane diastolic SR Ca^2+^ release. Therefore, under basal conditions mitochondrial Ca^2+^ fluxes modulate SR Ca^2+^ cycling via an effect on SR Ca^2+^ loading, and on this basis mitochondrial Ca^2+^ flux is indirectly linked to the SANC spontaneous AP firing rate.

## Materials and Methods

### Single SANC Isolation, Cell Contraction and Electrophysiological Recordings

Single, spontaneously beating SANC were isolated from New Zealand White rabbit hearts as previously described [13], using protocols approved by the Animal Care and Use Committee of the National Institutes of Health (protocol #034LCS2013). The cell contraction rate and Ca^2+^ cycling were studied in Tyrode solution at 35±0.5°C, with the following composition (in mM): 140 NaCl, 5.4 KCl, 2 MgCl_2_, 5 HEPES, 1.8 CaCl_2_, and 5.5 Glucose, and was titrated to pH 7.4 with NaOH. Cells were imaged with an LSM-510 inverted confocal microscope using a 63×/1.4 N.A. oil immersion lens (Carl Zeiss). Linescan images (using 633 nm He-Ne laser excitation, 512×1 pixels at 21.5 pixel/µm and 0.8 ms/line), were recorded with a scan line oriented along the short axis of the cell to quantify the spontaneous AP induced contraction rate. Cell contraction measurements were recorded for 15 min under control conditions and following drug application for times specified. Spontaneous action potentials were measured via a perforated patch-clamp technique with 35 µM β-escin (Sigma) added to the pipette solution that contained (in mM): 120 K-gluconate, 5 NaCl, 5 MgATP, 5 NaATP, 5 HEPES and 20 KCl, pH 7.2 with KOH. Action potentials were recorded using an Axopatch-200 B patch-clamp amplifier (Axon Instruments, Foster City, CA). For both cell contraction and electrophysiological recordings cells from at least 5 rabbits were used.

### Calibration and Measurements of Ca^2+^-Dependent Fluorescence Signals

Intracellular Ca^2+^ was measured with calibrated Indo-1 fluorescence to assess cytosolic Ca^2+^ signals. SANC were placed in a chamber on the inverted fluorescence microscope stage (Zeiss IM-35) and were loaded with 14 µM Indo-1 AM (Molecular Probes) for 15-min at room temperature, and subsequently superfused with Tyrode’s solution for 20-min to remove excess indicator and allow full de-esterification as the bath temperature slowly was increased to 35±0.5°C. The apparatus to detect Indo-1 fluorescence is as described previously [9], except that a 63x/1.4 N.A. oil UV fluorglycerin-immersion objective (Zeiss) was used. The Indo signals were corrected for background and autofluorescence by subtracting averaged signals from cells not loaded with Indo-1 (n = 5), determined in every experiment. [Ca^2+^]_i_ was calculated according to the equation [Ca^2+^]_i_ = βxK_d_x(R-R_min_)/(R_max_-R), using a K_d_ of 844 nM. The average R_min_ (minimal ratio), R_max_ (maximal ratio), and β (the ratio of maximal and minimal I_490_) for the fluorescence system were determined by sequential exposure of SANC to a high potassium, zero- Ca^2+^ solution (in mM: 132 KCl, 10 Hepes, 2 MgCl_2_, pH 7.2 with KOH) containing metabolic inhibitors (10 mM 2-deoxyglucose and 100 µM 2,4-dinitrophenol) (2), the same solution with 1 mM EGTA and 20 µM ionmycin (for R_min_). Measurements were taken when the fluorescence at both wavelengths reached stable values, and (3) high Ca^2+^ Tyrode’s solution (5 mM Ca^2+^ instead of EGTA) was used for determining R_max_. Average R_max_, R_min_ and β were 1.9±0.16, 0.9±0.01, and 2.1±0.6, respectively (n = 10). The method limitation derived from: 1) a change in pH from the calibrated solution (pH = 7.4 in bath solution compare to 7.2 see above), 2) imprecision of K_d_ of Indo-1 and 3) partial quenching of mitochondrial Ca^2+^ by Mn^2+^ affect the calculated [Ca^2+^]_m_. For Ca^2+^ recordings cells from at least 5 rabbits were used.

### Confocal Imaging for AP Triggered Spontaneous Local Ca^2+^ Releases

Ca^2+^ cycling into and out of the cytosol was measured with Flou-4 AM (Molecular Probes) to assess AP triggered Ca^2+^ release, spontaneous LCRs, and caffeine induced SR Ca^2+^ release. SANC were loaded with 5 µM Flou-4 AM for 20 min at room temperature, and subsequently superfused with Tyrode’s solution at 35±0.5°C. The Ca^2+^ fluorescence was imaged by a LSM510 confocal microscope (see above) using a 40x/1.3 N.A. oil immersion lens. Cells were excited with the 488 nm laser line of an Ar laser, and fluorescence emission was collected with LP 505 nm, with the pinhole set to obtain no more than 5 µm optical slice (512×1 pixels at 14.9 pixel/µm and 2 ms/line). All images were recorded with a scan line oriented along the long axis of the cell, close to the sarcolemmal membrane, and processed with IDL software or Matlab.

The AP induced Ca^2+^ transient was expressed as a peak value (F) normalized to minimal fluorescence (F_0_). The amplitude of individual LCRs was also expressed as a peak value (F) normalized to minimal fluorescence (F_0_). LCR spatial size (FWHM) was indexed as the full width at half-maximum amplitude. LCR duration (FDHM) was characterized as the full duration at half-maximum amplitude. The number of LCRs was normalized per 100 µm of the linescan image and during a 1s time interval. The integral of Ca^2+^ of an individual LCR was estimated as follows: FWHMxFDHMx(F/F_0_-1)/2. The total Ca^2+^ released by LCR ensemble was calculated as the sum of the signal masses of diastolic LCRs that occurred between AP-induced Ca^2+^ transients. The SR Ca^2+^ content was estimated by the amplitude of the Ca^2+^ transient induced by a brief rapid application of caffeine (20 mM, 1s) onto the cell by pressure-ejection via a nearby pipette, as described previously [5]. For Ca^2+^ recordings cells from at least 5 rabbits were used.

### Cell Permeabilization

SANC were permeabilized with 5 or 25 µM digitonin at 35±0.5°C with solution containing (in mM): 100 K aspartate, 25 KCl, 10 NaCl, 3 MgATP, 0.8 MgCl2, (free [Mg^2+^] ∼1 mM), 20 HEPES, 0.5 EGTA, 10 phosphocreatine, and 5 U/ml creatine phosphokinase; pH 7.2 with KOH.

### Immunostaining

SANC were incubated with 500 nM Mitotracker Orange (MT; Molecular Probes), for 1 h at 37°C, fixed with 4% formaldehyde in phosphate buffer (PBS) and permeabilized with 1% Triton X-100/PBS. Nonspecific cross-reactivity was blocked by incubating the samples overnight in solution containing: 2% BSA/PBS, 5% donkey serum, 0.01% NaN_3_, and 0.2% Triton. SANC were then incubated with anti-SERCA2 antibody (1∶100, IgG1, clone IID8; Affinity BioReagents, Golden, CO) at 4°C overnight. This was followed by labeling with a Cy5-conjugated secondary antibody (1∶1000, anti-mouse, Jackson ImmunoResearch) incubation. The fluorescence was imaged by a LSM510 confocal microscope (see above) using a 40x/1.3 N.A oil immersion lens. The Cy5 and MT fluorophores were excited with 633 and 543 nm He-Ne lasers, respectively.

### Western Blot of Phospholamban

SANC suspensions were divided equally into 4 aliquots: the first aliquot was treated with 2 µM Ru360, the second with 1 µM CGP-37157, the third with 1 µM isoproterenol; and the forth was used as a control. Cells were incubated at 36°C for 10 min. After the treatment, samples were centrifuged and the pellets were frozen in liquid nitrogen. Pelleted cells were resuspended in RIPA lysis buffer (150 mM NaCl, 1% Triton X-100, 0.5% sodium deoxycholate, 0.1% SDS and 50 mM Tris, with pH adjusted to 8) that also included a standard mammalian protease inhibitor cocktail (Sigma) and phosphatase inhibitors 1 mM NaF, 2 mM Na_3_VO_4_. Total protein concentration was determined using a standard, commercially available protein quant kit (GE Healthcare, 2D protein Quant kit). The accuracy of protein quantification was verified on the 4–12% Bis-Tris polyacrylamide SDS-PAGE gel (Invitrogen) labeled with the fluorescent Sypro Ruby gel stain (Invitrogen). The total integral of fluorescence intensities for each protein lane was calculated using ImageQuant5 software (GE Healthcare). 5-15 µg of proteins from each aliquot were mixed with the NuPage sample buffer and DTT (10 mM final), and resolved on 4–12% Bis-Tris polyacrylamide SDS-PAGE gel using standard MES/SDS running buffer. Proteins were transferred for 25 min to the activated PVDF membrane (0.45 µm, Molecular Probes) using standard semidry transfer unit (Bio-Rad) in NuPage transfer buffer (Bicine 25 mM, Bis-tris 25 mM EDTA 1.0 mM, Methanol 20%, pH 7.2, Invitrogen). PVDF membranes with transferred proteins were blocked overnight in 5% nonfat milk (Bio-Rad), and resuspended in TBS-T buffer (50 mM Tris-HCl; 500 mM NaCl, 1% Tween, pH 7.5).

To detect total phospholamban the first membrane was incubated over-night with a total anti-PLB monoclonal phospholamban antibody (1∶7000, Badrilla) and incubated 1 h at room temperature with goat anti-mouse conjugated antibodies (1∶5000, Dako). To detect phospholamban phosphorylation, the second and the third membranes were incubated over-night with anti-P-Ser-16 rabbit polyclonal antibody (1∶2000, Badrilla) or the anti- P-Thr-17 rabbit antibody (1∶2000, Badrilla), and incubated for 1 h at room temperature with polyclonal goat anti-rabbit secondary antibody (1∶5000, Dako). To detect actin, the membranes were incubated over-night with anti-actin goat polyclonal antibodies (1∶3000, Santa Cruz), and incubated for 1 h at room temperature with donkey anti-goat-IgG-HRP secondary antibody (1∶5000, Santa Cruz). Membranes were developed for 5 min using SuperSignal West Pico (or Dura) Chemiluminescent Substrate (Thermo Fisher Scientific) and visualized on the HyBlot CL autoradiography film using a series of sequential exposure times (Denville Scientific Inc.) to obtain an optimum signal intensity.

### Drugs

CGP-37157, a mitochondrial Na^+^-Ca^2+^ exchanger inhibitor, and Ru360, an inhibitor of Ca^2+^ flux into the mitochondria, were purchased from EMD Chemicals; Isoproterenol, digitonin, saponin, ionmycin, cesium chloride, cyclopiazonic acid, caffeine and MnCl_2_ were purchased from Sigma.

### Statistical Analysis

Data are presented as mean±SEM. When the means of paired samples were to be compared, a paired t-test was employed. For multiple pharmacologic treatments, a linear mixed-effects model with Dunnett’s method to adjust p-values was used. This model accounts for repeated measurements on the same preparation while allowing testing for differences among pharmacological perturbations. *P<*0.05 was taken to indicate statistical significance.

## Supporting Information

Figure S1 **Validation of compartmentatal indo-quenching.** (A–B) Low concentration of digitonin (5 µmol/L) does not permeabilize mitochondrial membrane, however high concentration of digitonin (25 µmol/L) does permeabilize mitochondrial membrane (C–D) visualized by 125 nmol/L tetramethylrhodamine methyl ester (TMRM).
(TIF)

Figure S2 **Representative examples of Indo-1 fluorescence.** Mn^2+^ (50 µmol/L) quenching of the cytosolic indo-1 fluorescence ratio 410/490 in the presence of Ru360 (upper panel) and CGP-37157 (lower panel).
(TIF)

Figure S3 **Co-immunoelabeling of SERCA2 and part of the mitochondrial mass in SANC, visualized by anti-SERCA2 antibody and Mitotracker orange (MT) staining, respectively.**
(TIF)

Figure S4 **Western blots of phospholamban phosphorylation.** (A) Representative examples of phospholamban phosphorylated at serine-16 site (PKA site) and total phospholamban in the basal state and following Ru360 (2 µmol/L), CGP-37157 (1 µmol/L) or isoproterenol (1 µmol/L). Actin is used as a protein loading control. (B) Fluorescent Sypro Ruby gel stain to validate the accuracy of protein loading. (C) Average phosphorylation ratio (n = 5) of phospholamban phosphorylated at serine-16 site to protein loading. *p<0.05 vs. control.
(TIF)

Figure S5 **Average time-dependent change in the rate of AP induced contractions in the presence of CsCl and CGP-37157 (n = 7) or CsCl and Ru360 (n = 7).**
(TIF)

Figure S6 **Mitochondrial-SR numerical model.** The extended coupled clock numerical model simulations of the effect of specific inhibition of Ca influx into or efflux from mitochondria in intact SANC on (A) Ca^2+^ in network SR, (B) Ca^2+^ in junctional SR, (C) Ca^2+^ release flux from the SR, and (D) Ca^2+^ in the sub membrane space.
(TIF)

Table S1 (DOC)

Table S2 (DOC)

Text S1 (DOC)
