# Supplementary figures and images for: Crosstalk between Mitochondrial and Sarcoplasmic Reticulum Ca2+ Cycling Modulates Cardiac Pacemaker Cell Automaticity
Source: PLoS One. 2012 May 29;7(5):e37582. doi: 10.1371/journal.pone.0037582 (PMC3362629; doi:10.1371/journal.pone.0037582)

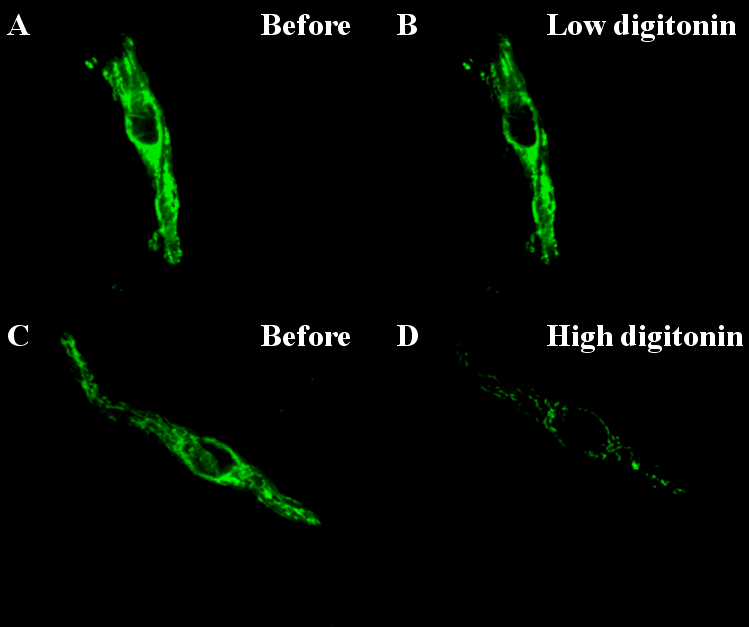

Supplement: Figure S1 — Validation of compartmentatal indo-quenching. (A–B) Low concentration of digitonin (5 µmol/L) does not permeabilize mitochondrial membrane, however high concentration of digitonin (25 µmol/L) does permeabilize mitochondrial membrane (C–D) visualized by 125 nmol/L tetramethylrhodamine methyl ester (TMRM). (TIF) [file pone.0037582.s001.tif]

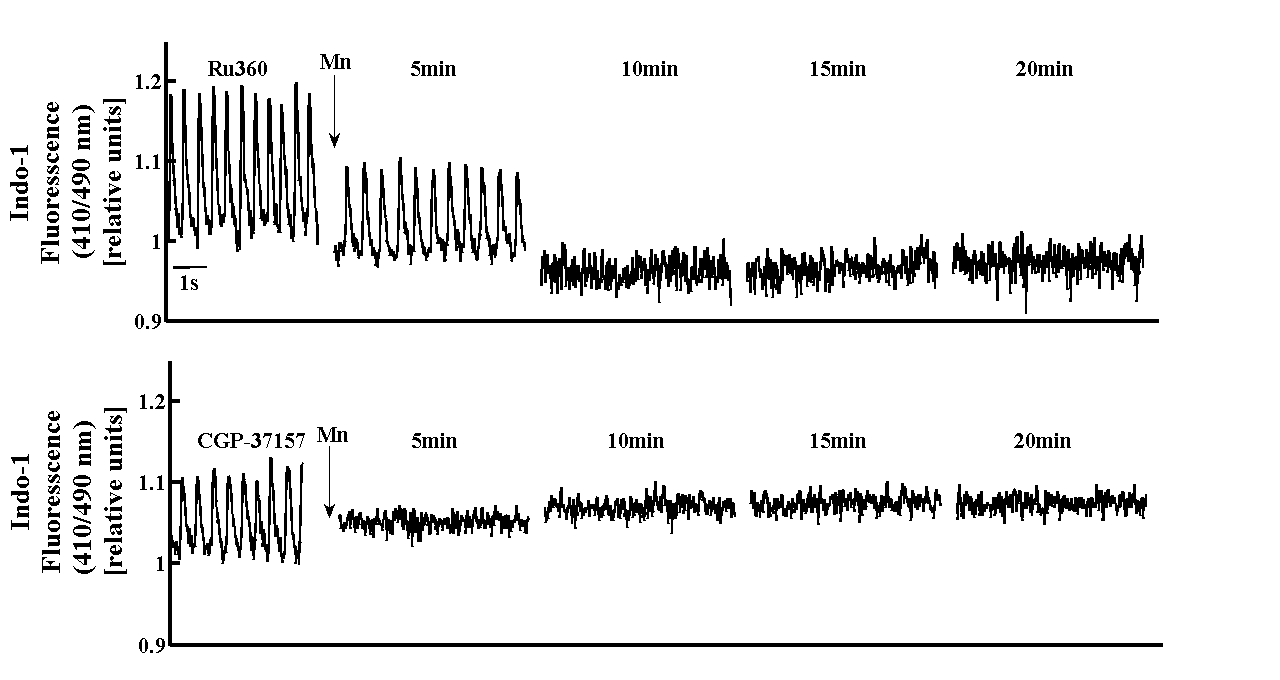

Supplement: Figure S2 — Representative examples of Indo-1 fluorescence. Mn2+ (50 µmol/L) quenching of the cytosolic indo-1 fluorescence ratio 410/490 in the presence of Ru360 (upper panel) and CGP-37157 (lower panel). (TIF) [file pone.0037582.s002.tif]

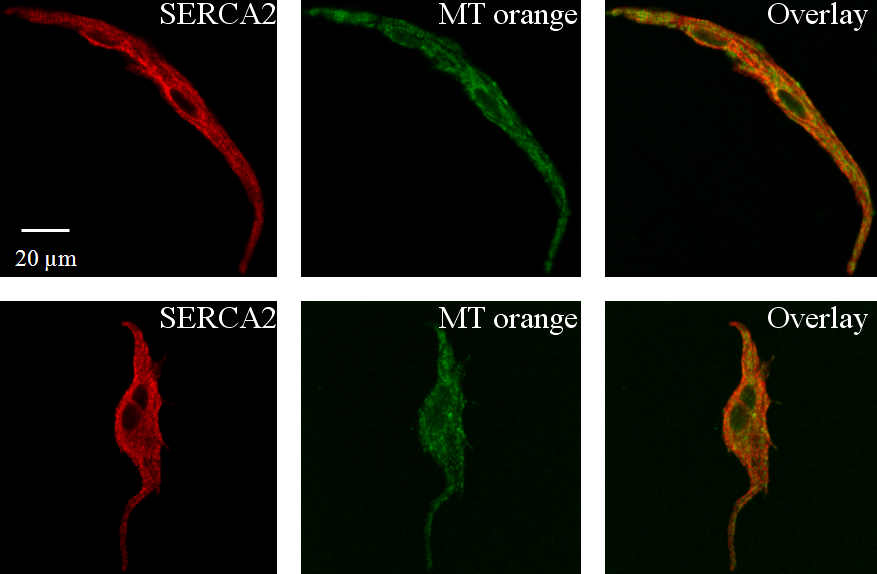

Supplement: Figure S3 — Co-immunoelabeling of SERCA2 and part of the mitochondrial mass in SANC, visualized by anti-SERCA2 antibody and Mitotracker orange (MT) staining, respectively. (TIF) [file pone.0037582.s003.tif]

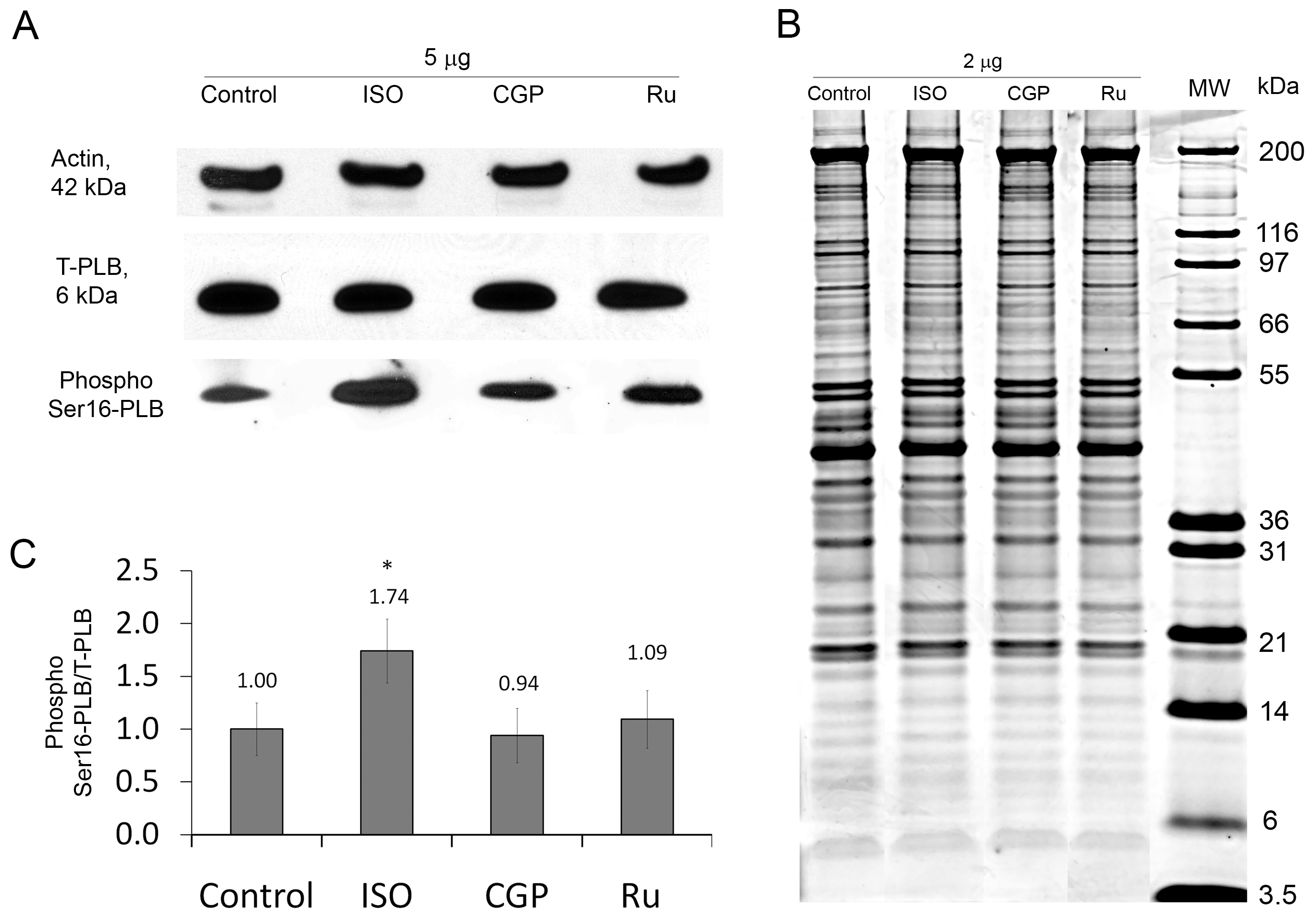

Supplement: Figure S4 — Western blots of phospholamban phosphorylation. (A) Representative examples of phospholamban phosphorylated at serine-16 site (PKA site) and total phospholamban in the basal state and following Ru360 (2 µmol/L), CGP-37157 (1 µmol/L) or isoproterenol (1 µmol/L). Actin is used as a protein loading control. (B) Fluorescent Sypro Ruby gel stain to validate the accuracy of protein loading. (C) Average phosphorylation ratio (n = 5) of phospholamban phosphorylated at serine-16 site to protein loading. *p<0.05 vs. control. (TIF) [file pone.0037582.s004.tif]

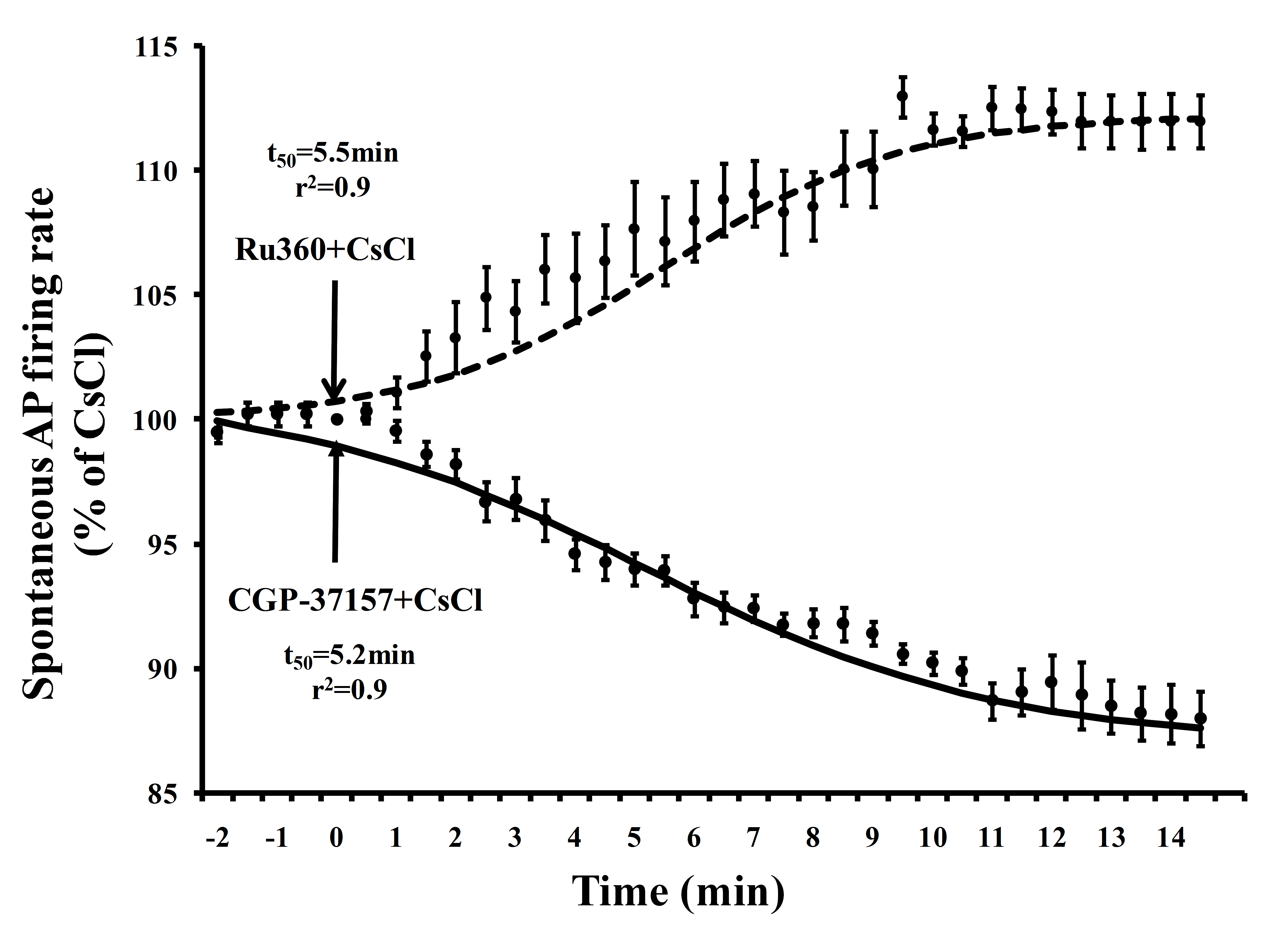

Supplement: Figure S5 — Average time-dependent change in the rate of AP induced contractions in the presence of CsCl and CGP-37157 (n = 7) or CsCl and Ru360 (n = 7). (TIF) [file pone.0037582.s005.tif]

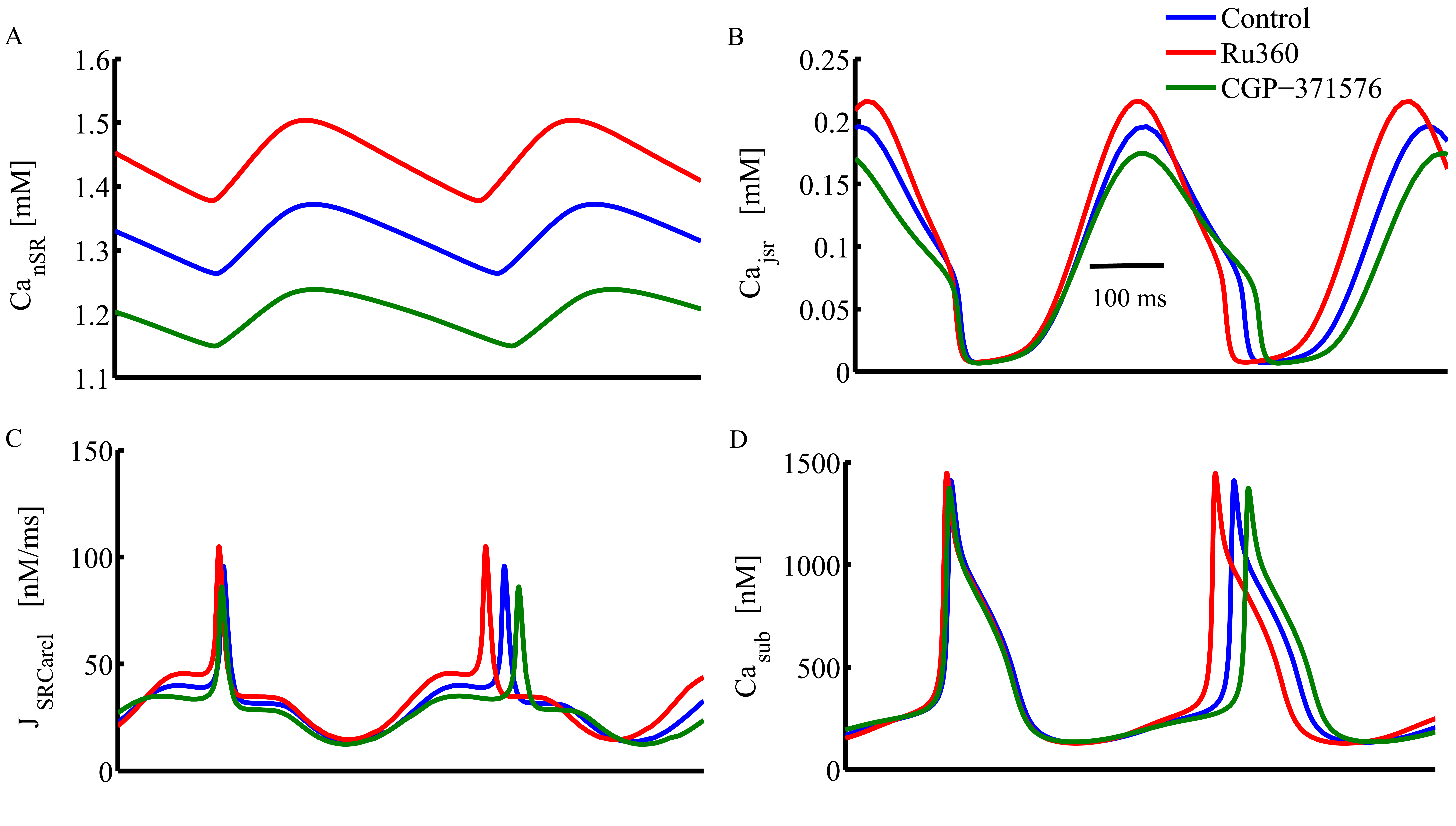

Supplement: Figure S6 — Mitochondrial-SR numerical model. The extended coupled clock numerical model simulations of the effect of specific inhibition of Ca influx into or efflux from mitochondria in intact SANC on (A) Ca2+ in network SR, (B) Ca2+ in junctional SR, (C) Ca2+ release flux from the SR, and (D) Ca2+ in the sub membrane space. (TIF) [file pone.0037582.s006.tif]
