# Supplementary material for: Crosstalk between Mitochondrial and Sarcoplasmic Reticulum Ca2+ Cycling Modulates Cardiac Pacemaker Cell Automaticity
Source: PLoS One. 2012 May 29;7(5):e37582. doi: 10.1371/journal.pone.0037582 (PMC3362629; doi:10.1371/journal.pone.0037582)
Supplement: Table S1 — (DOC) [file pone.0037582.s007.doc]

| **Fixed ion concentrations** | | | | | | |
| --- | --- | --- | --- | --- | --- | --- |
| **Symbol** | | **Value** | | **Units** | | **Description** |
| Ca_o_ | | 2 | | mM | | Extracellular [Ca^2+^]. |
| K_o_ | | 5.4 | | mM | | Extracellular [K^+^]. |
| K_i_ | | 140 | | mM | | Intracellular [K^+^]. |
| Na_o_ | | 140 | | mM | | Extracellular [Na^+^]. |
| Na_i_ | | 10 | | mM | | Intracellular [Na^+^]. |
| Mg_i_ | | 2.5 | | mM | | Intracellular [Mg^2+^]. |
| **Cell compartments** | | | | | | |
| **Symbol** | | **Value** | | **Units** | | **Description** |
| *C*_m_ | | 32 | | pF | | Cell electric capacitance. |
| *V*_cell_ | | 3.5185838 | | pL | | Cell volume. |
| *V*_sub_ | | 0.035097874 | | pL | | Submembrane space volume. |
| *V*_jSR_ | | 0.0042 | | pL | | Volume of junctional SR (Ca^2+^ release store). |
| *V*_i_ | | 1.5835 | | pL | | Myoplasmic volume. |
| *V*_nSR_ | | 0.0408 | | pL | | Volume of network SR (Ca^2+^ uptake store). |
| *V*_myto_ | | 0.6334 | | pL | | Mitochondrial volume. |
| F | | 96485 | | C/M | | Faraday constant. |
| T | | 310 | | K˚ | | Absolute temperature for 37˚C. |
| R | | 8.3144 | | J/(M·K˚) | | Universal gas constant. |
| *E*_CaL_ | | 45 | | mV | | Apparent reversal potential of *I*_CaL_. |
| *E*_CaT_ | | 45 | | mV | | Apparent reversal potential of *I*_CaT_. |
| *E*_st_ | | 37.4 | | mV | | Apparent reversal potential of *I*_st_. |
| **Membrane Ca^2+^ current parameters** | | | | | | |
| **Symbol** | **Value** | | **Units** | **Description** | | |
| *g*_CaL,max_ | 0.52 | | nS/pF | Maximal L-type Ca^2+^current conductance. | | |
| *V*_½,d_ | -13.5 | | mV | Steady-state activation parameters | | |
| *K*_d_ | 6 | | mV |  | | |
| *V*_½,f_ | -35 | | mV | Steady-state inactivation parameters | | |
| *K*_f_ | 7.3 | | mV |  | | |
| *K*_mfCa_ | 0.00035 | | mM | Dissociation constant of Ca^2+^ -dependent *I*_CaL_ inactivation. | | |
| *α*_fCa_ | 0.021 | | ms^-1^ | Ca^2+^ dissociation rate constant for *I*_CaL_ | | |
| *g*_CaT,max_ | 0.1832 | | nS/pF | Maximal T-type Ca^2+^ current conductance. | | |
| *g*_bCa_ | 0.0006 | | nS/pF | Maximal background Ca^2+^ current conductance. | | |
| **Other membrane current parameters** | | | | | | |
| **Symbol** | **Value** | | **Units** | **Description** | | |
| *g*_If,max_ | 0.15 | | nS/pF | Maximal hyperpolarization-activated current conductance. | | |
| *V*_If,1/2_ | -64 | | mV | Half activation voltage for *I*_f_ current in the basal state. | | |
| *g*_st,max_ | 0.00001 | | nS/pF | Maximal Sustained inward current conductance. | | |
| *g*_Kr,max_ | 0.0811397 | | nS/pF | Maximal delayed rectifier K current rapid component conductance. | | |
| *g*_Krsmax_ | 0.0259 | | nS/pF | Maximal delayed rectifier K current slow component conductance. | | |
| *g*_to,max_ | 0.252 | | nS/pF | Maximal 4-aminopyridine sensitive transient K current conductance. | | |
| *g*_sus,max_ | 0.02 | | nS/pF | Maximal 4-aminopyridine sensitive sustained K^+^ current conductance. | | |
| *I*_NaK,max_ | 2.88 | | pA/pF | Maximal Na/K pump current conductance. | | |
| *K*_mKp_ | 1.4 | | mM | Half-maximal *K*_o_ for *I*_NaK_. | | |
| *K*_mNap_ | 14 | | mM | Half-maximal *Na*_i_ for *I*_NaK_. | | |
| *g*_bNa_ | 0.00486 | | nS/pF | Maximal background Na^+^current conductance. | | |
| **Na^+^/Ca^2+^ exchanger current parameters** | | | | | | |
| **Symbol** | **Value** | | **Units** | **Description** | | |
| *k*_NCX_ | 225 | | pA/pF | Maximal Na^+^/Ca^2+^ exchanger current conductance. | | |
| *K*_1ni_ | 395.3 | | [] | Intracellular Na^+^ binding to first site on NCX. | | |
| *K*_2ni_ | 2.289 | | [] | Intracellular Na^+^ binding to second site on NCX. | | |
| *K*_3ni_ | 26.44 | | [] | Intracellular Na^+^ binding to third site on NCX. | | |
| *K*_1no_ | 1628 | | [] | Extracellular Na^+^ binding to first site on NCX. | | |
| *K*_2no_ | 561.4 | | [] | Extracellular Na^+^ binding to second site on NCX. | | |
| *K*_3no_ | 4.663 | | [] | Extracellular Na^+^ binding to third site on NCX. | | |
| *K*_ci_ | 0.0207 | | [] | Intracellular Ca^2+^ binding to NCX transporter. | | |
| *K*_co_ | 3.663 | | [] | Extracellular Ca^2+^ binding to NCX transporter. | | |
| *K*_cni_ | 26.44 | | [] | Intracellular Na^+^ and Ca^2+^ simultaneous binding to NCX. | | |
| *Q*_ci_ | 0.1369 | | [] | Intracellular Ca^2+^ occlusion reaction of NCX. | | |
| *Q*_co_ | 0 | | [] | Extracellular Ca^2+^ occlusion reaction of NCX. | | |
| *Q*_n_ | 0.4315 | | [] | Na^+^ occlusion reactions of NCX. | | |
| **Ca^2+^ flux parameters** | | | | | | |
| **Symbol** | **Value** | | **Units** | **Description** | | |
| *τ*_difCa_ | 0.04 | | ms | Time constant of Ca^2+^ diffusion from the submembrane to myoplasm. | | |
| *τ*_tr_ | 40 | | ms | Time constant for Ca^2+^ transfer from the network to junctional SR. | | |
| *K*_up_ | 0.6·10^-3^ | | mM | Half-maximal Ca_i_ for Ca^2+^ uptake in the network SR. | | |
| *P*_up,basal_ | 0.012 | | mM /ms | Rate constant for Ca^2+^ uptake by the Ca^2+^ pump in the network SR. | | |
| *P*_up,Ru360_ | 0.0132 | | mM /ms | -”- | | |
| *P*_up,CGP_ | 0.0108 | | mM /ms | -”- | | |
| *k*_oCa_ | 10 | | mM ^-2^· ms^-1^ | Ryanodine channel parameters. | | |
| *k*_om_ | 0.06 | | ms^-1^ |  | | |
| *k*_iCa_ | 0.5 | | mM ^-1^· ms^-1^ |  | | |
| *k*_im_ | 0.005 | | ms^-1^ |  | | |
| *EC*50_SR | 0.45 | | mM |  | | |
| *k*_s_ | 400·10^3^ | | ms^-1^ |  | | |
| *MaxSR* | 13 | | [] |  | | |
| *MinSR* | 1 | | [] |  | | |
| *HSR* | 3 | | [] |  | | |
| **Natural buffering of Ca^2+^ and Mg^2+^parameters** | | | | | | |
| **Symbol** | **Value** | | **Units** | | **Description** | |
| *k*_bCM_ | 0.542 | | ms^-1^ | | Ca^2+^ dissociation constant for calmodulin. | |
| *k*_bCQ_ | 0.445 | | ms^-1^ | | Ca^2+^ dissociation constant for calsequestrin. | |
| *k*_bTC_ | 0.446 | | ms^-1^ | | Ca^2+^ dissociation constant for the troponin-Ca^2+^ site. | |
| *k*_bTMC_ | 0.00751 | | ms^-1^ | | Ca^2+^ dissociation constant for the troponin-Mg^2+^ site. | |
| *k*_bTMM_ | 0.751 | | ms^-1^ | | Mg^2+^ dissociation constant for the troponin-Mg^2+^ site. | |
| *k*_fCM_ | 227.7 | | mM ^-1^· ms^-1^ | | Ca^2+^ association constant for calmodulin. | |
| *k*_fCQ_ | 0.534 | | mM ^-1^· ms^-1^ | | Ca^2+^ association constant for calsequestrin. | |
| *k*_fTC_ | 88.8 | | mM /ms | | Ca^2+^ association constant for troponin. | |
| *k*_fTMC_ | 227.7 | | mM /ms | | Ca^2+^ association constant for the troponin-Mg^2+^ site. | |
| *k*_fTMM_ | 2.277 | | mM /ms | | Mg^2+^ association constant for the troponin-Mg^2+^ site. | |
| *TC*_tot_ | 0.031 | | mM | | Total concentration of the troponin-Ca^2+^ site. | |
| *TMC*_tot_ | 0.062 | | mM | | Total concentration of the troponin-Mg^2+^ site. | |
| *CQ*_tot_ | 10 | | mM | | Total calsequestrin concentration. | |
| *CM*_tot_ | 0.045 | | mM | | Total calmodulin concentration. | |
| **Mitochondrial Ca^2+^ parameters** | | | | | | |
| **Symbol** | **Value** | | **Units** | | **Description** | |
| β_Ca_ | 0.01 | | [] | | The fraction of Ca^2+^ that binds to Ca^2+^ buffers in the mitochondria. | |
| P_Ca_ | 1.1672 | | ms^-1^ | | Uniporter Ca^2+^ permeability. | |
| P_Ca,Ru360_ | 0.0817 | | ms^-1^ | | Uniporter Ca^2+^ permeability. | |
| ψ_m_ | 154.226 | | mV | | Mitochondrial membrane potential. | |
| α_m_ | 0.2 | | [] | | Mitochondrial activity coefficients. | |
| α_e_ | 0.341 | | [] | | Extramitochondrial activity coefficients. | |
| Q_mo_ | 0.024 | | mM/ms | | Na^+^-Ca^2+^ exchanger maximal velocity. | |
| *Q*_mo,CGP-37157_ | 0.017 | | mM/ms | | Na^+^-Ca^2+^ exchanger maximal velocity. | |
| K_ca,m_ | 0.003 | | mM | | Na^+^-Ca^2+^ exchanger Ca^2+^ affinity. | |
