# Supplementary material for: Crosstalk between Mitochondrial and Sarcoplasmic Reticulum Ca2+ Cycling Modulates Cardiac Pacemaker Cell Automaticity
Source: PLoS One. 2012 May 29;7(5):e37582. doi: 10.1371/journal.pone.0037582 (PMC3362629; doi:10.1371/journal.pone.0037582)
Supplement: Table S2 — (DOC) [file pone.0037582.s008.doc]

| **Initial values of state variables (Ca^2+^  cycling)** | | | |
| --- | --- | --- | --- |
| **Symbol** | **Value** | **Units** | **Description** |
| *Ca_i_* | 0.0001 | mM | [Ca^2+^] in myoplasm. |
| *Ca_sub_* | 0.000223 | mM | [Ca^2+^] in submembrane space. |
| *Ca*_jSR_ | 0.029 | mM | [Ca^2+^] in the junctional SR (jSR). |
| *Ca*_nSR_ | 1.35 | mM | [Ca^2+^] in the network SR (nSR). |
| *Ca*_m_ | 0.00005 | mM | [Ca^2+^] in mitochondria. |
| *f*_TC_ | 0.02 | [] | Fractional occupancy of the troponin-Ca^2+^ site by Ca^2+^ in myoplasm. |
| *f*_TMC_ | 0.22 | [] | Fractional occupancy of the troponin-Mg^2+^ site by Ca^2^ in myoplasm. |
| *f*_TMM_ | 0.69 | [] | Fractional occupancy of the troponin-Mg^2+^ site by Mg^2+^ in myoplasm. |
| *f*_CMi_ | 0.042 | [] | Fractional occupancy of calmodulin by Ca^2+^ in myoplasm. |
| *f*_CMs_ | 0.089 | [] | Fractional occupancy of calmodulin by Ca^2+^ in submembrane space. |
| *f*_CQ_ | 0.032 | [] | Fractional occupancy of calsequestrin by Ca^2+^ in junctional SR. |
| *R* | 0.7499955 | [] | RyR reactivated (closed) state. |
| *O* | 3.4·10^-6^ | [] | RyR open state. |
| *I* | 1.1·10^-6^ | [] | RyR inactivated state. |
| *RI* | 0.25 | [] | RyR RI state. |
| **Initial values of state variables (electrophysiology)** | | | |
| **Symbol** | **Value** | **Units** | **Description** |
| *V_m_* | -65 | mV | Membrane potential. |
| *d*_L_ | 0 | [] | *I*_CaL_ activation. |
| *f*_L_ | 1 | [] | *I*_CaL_ voltage-dependent inactivation. |
| *f*_Ca_ | 1 | [] | *I*_CaL_ Ca^2+^ dependent inactivation. |
| *p*_aF_ | 0 | [] | *I*_Kr_ fast activation. |
| *p*_aS_ | 0 | [] | *I*_Kr_ slow activation. |
| *p*_i_ | 1 | [] | *I*_Kr_ inactivation. |
| n | 0 | [] | I_Ks_ activation. |
| *x* | 1 | [] | *I*_f_ activation. |
| *d*_T_ | 0 | [] | *I*_CaT_ activation. |
| *f*_T_ | 1 | [] | *I*_CaT_ inactivation. |
| *q* | 1 | [] | *I*_to_ inactivation. |
| *r* | 0 | [] | *I*_to_ and *I*_sus_ activation. |
| q_a_ | 0 | [] | I_st_ activation. |
| q_i_ | 1 | [] | I_st_ inactivation. |
