# Supplementary material for: Crosstalk between Mitochondrial and Sarcoplasmic Reticulum Ca2+ Cycling Modulates Cardiac Pacemaker Cell Automaticity
Source: PLoS One. 2012 May 29;7(5):e37582. doi: 10.1371/journal.pone.0037582 (PMC3362629; doi:10.1371/journal.pone.0037582)
Supplement: Text S1 — (DOC) [file pone.0037582.s009.doc]

**On-Line Supplement**

**Crosstalk between mitochondrial and sarcoplasmic reticulum Ca^2+^ cycling modulates cardiac pacemaker cell automaticity**

**Yaniv. Mitochondrial-SR Ca^2+^ cycling in pacemaker cells**

**Yael Yaniv, Harold A. Spurgeon, Alexey E. Lyashkov,**

**Dongmei Yang, Bruce D. Ziman, Victor A. Maltsev**

**and Edward G. Lakatta**

*Laboratory of Cardiovascular Science, Gerontology Research Center, Intramural Research Program, National Institute on Aging, NIH, Baltimore, Maryland, USA.*

**Numerical model**

Our numerical model is based the surface membrane electrogenic molecule currents and SR equations described by the Maltsev and Lakatta coupled-clock numerical model,[1] and includes an additional mitochondrial Ca^2+^ flux description, to simulate, for the first time, the effect of mitochondrial Ca^2+^ flux on the SR Ca^2+^ flux. The present modified coupled-clock numerical model includes 27 first-order differential equations. The model parameters and equations are based on Maltsev and Lakatta coupled-clock numerical model[1] and provided here for the reader’s convenience.

1. **Mitochondria fluxes**

**Mitochondrial Ca^2+^ fluxes**

We assume that the majority of Ca^2+^ enters through the uniporter.[2,3] The uniporter behaves as an ion-channel and therefore the flux through it can be described by Goldman-Hodgkin-Katz equation:[2,4]

$j_{uni}=P_{Ca}\frac{z_{Ca}\Psi_{m}F}{RT}\frac{\alpha_{m}[Ca^{2+}]_{m}\exp\left[\frac{-z_{Ca}\Psi_{m}F}{RT}\right]-\alpha_{e}[Ca^{2+}]_{i}}{\exp\left[\frac{-z_{Ca}\Psi_{m}F}{RT}\right]-1}$ (1)

where $P_{Ca}$is the permeability of the Ca^2+^ uniporter, $\Psi_{m}$ is the mitochondrial membrane potential, $\alpha_{m}$ and $\alpha_{e}$are the mitochondrial and extramitochondrial activity coefficients. We assume a constant mitochondrial membrane potential.[5]

Ca^2+^ outflow from the mitochondria via the Na^+^-Ca^2+^ exchanger (NCX_m_). The Na^+^-Ca^2+^ exchanger flux is described by:[4]

$j_{NaCa,m}=Q_{mo}\frac{[Ca^{2+}]_{m}}{K_{Ca,m}+[Ca^{2+}]_{m}}$ (2)

The coefficient $Q_{mo}$depends on NCX_m_ maximal velocity, NCX_m_ Na^+^ affinity and intracellular Na^+^ concentration. $K_{Ca,m}$is the NCX_m_ Ca^2+^ affinity

Therefore, the balance equation of [Ca^2+^]_m_ is described by

$\frac{d[Ca^{2+}]_{m}}{dt}=\beta_{Ca}(j_{uni}-j_{NaCa,m})$ (3)

where $\beta_{Ca}$is a constant describing the fraction of Ca^2+^ that binds to Ca^2+^ buffers in the mitochondria. $Q_{mo}$,$P_{Ca}$ and $\beta_{Ca}$were based on Nguyen et al.[2] and modified to fit to our experimental data.

1. **Ionic currents**

**Gating variable**

$\frac{dx}{dt}=\frac{x_{\infty}-x}{\tau_{x}}$ (4)

x: $d_{L},f_{L},f_{Ca},d_{T},f_{T},p_{aF},p_{aS},p_{i},n,q,r,y,q_{a},q_{i}$

where $\tau_{x}$is the time constant for a gating variable *x_i_* and $x_{\infty}$is the steady-state curve for the gating variable *x_i_.*

**L-type Ca^2+^ current (*I*_CaL_)**

The channel current formulations are based Kurata et al. model.[6]

$I_{CaL}=C_{m}g_{CaL\max}(V_{m}-E_{CaL})d_{L}f_{L}f_{Ca}$ (5)

$d_{L,\infty}=\frac{1}{(1+\exp[-(V_{m}-V_{1/2,d})/K_{d}])}$ (6)

$f_{L,\infty}=\frac{1}{(1+\exp[(V_{m}-V_{1/2,f})/K_{f}])}$ (7)

$\alpha_{dL}=\frac{-0.02839(V_{m}+35)}{(\exp[-(V_{m}+35)/2.5]-1)}-\frac{0.0849V_{m}}{(\exp(-V_{m}/4.8)-1)}$ (8)

$\beta_{dL}=\frac{0.01143(V_{m}-5)}{(\exp[(V_{m}-5)/2.5]-1)}$ (9)

$\tau_{dL}=\frac{1}{\alpha_{dL}+\beta_{dL}}$ (10)

$\tau_{fL}=257.1\exp\{-[(V_{m}+32.5)/13.9]^{2}\}+44.3$ (11)

$f_{Ca,\infty}=\frac{K_{mfCa}}{K_{mfCa}+Ca_{sub}}$ (12)

$\tau_{fCa}=\frac{f_{Ca,\infty}}{\alpha_{fCa}}$ (13)

**T-type Ca^2+^ current (*I*_CaT_)**

The channel current formulations are based on Demir et al.[7] model and were modified by Kurata et al. model.[6]

$I_{CaT}=C_{m}g_{CaT,\max}(V_{m}-E_{CaT})d_{T}f_{T}$ (14)

$d_{T,\infty}=\frac{1}{1+\exp(-(V_{m}+26.3)/6)}$ (15)

$f_{T,\infty}=\frac{1}{1+\exp((V_{m}+61.7)/5.6)}$ (16)

$\tau_{dT}=\frac{1}{1.068\exp((V_{m}+26.3)/30)+1.068(-(V_{m}+26.3)/30)}$ (17)

$\tau_{dT}=\frac{1}{0.0153\exp(-(V_{m}+61.7)/83.3)+0.015((V_{m}+61.7)/15.38)}$ (18)

**Rapidly activating delayed rectifier K^+^ current (*I*_Kr_)**

The channel current formulations are based on Kurata et al. model.[6]

$I_{Kr}=C_{m}g_{Kr,\max}(V_{m}-E_{K})(0.6p_{aF}+0.4p_{aS})p_{i}$ (19)

$E_{K}=\frac{RT}{F}\ln(\frac{K_{o}}{K_{i}})$ (20)

$p_{aF,\infty}=p_{aS,\infty}=\frac{1}{1+\exp(-(V_{m}+23.2)/10.6)}$ (21)

$p_{i,\infty}=\frac{1}{1+\exp((V_{m}+28.6)/17.1)}$ (22)

$\tau_{paF}=\frac{0.84655354}{0.0372\exp(V_{m}/15.9)+0.00096\exp(-V_{m}/22.5)}$ (23)

$\tau_{paS}=\frac{0.84655354}{0.0042\exp(V_{m}/17)+0.00015\exp(-V_{m}/21.6)}$ (24)

$\tau_{pi}=\frac{1}{0.1\exp(-V_{m}/54.645)+0.656\exp(V_{m}/106.157)}$ (25)

**Slow activating delayed rectifier K^+^ current (*I*_Ks_)**

The channel current formulations are based on Zhang et al. model.[8]

$I_{Ks}=C_{m}g_{Ks,\max}(V_{m}-E_{Ks})n^{2}$ (26)

$E_{Ks}=\frac{RT}{F}\ln(\frac{K_{0}+0.12Na_{0}}{K_{i}+0.12Na_{i}})$ (27)

$\alpha_{n}=\frac{0.014}{1+\exp(-(V_{m}-40)/9)}$ (28)

$\beta_{n}=0.001\exp(-V_{m}/45)$ (29)

$n_{\infty}=\frac{\alpha_{n}}{\alpha_{n}+\beta_{n}}$ (30)

$\tau_{n}=\frac{1}{\alpha_{n}+\beta_{n}}$ (31)

**4-aminopyridine-sensitive currents (*I*_4AP_ =*I*_to_ *+ I*_sus_)**

The channel current formulations are based on Zhang et al. model.[8]

$I_{to}=C_{m}g_{to,\max}(V_{m}-E_{K})qr$ (32)

$I_{sus}=C_{m}g_{sus,\max}(V_{m}-E_{K})r$ (33)

$q_{\infty}=\frac{1}{1+\exp((V_{m}+49)/13)}$ (34)

$r_{\infty}=\frac{1}{1+\exp(-(V_{m}-19.3)/15)}$ (35)

$\tau_{q}=\frac{39.102}{0.57\exp(-0.08(V_{m}+44))+0.065\exp(0.1(V_{m}+45.93))}+6.06$ (36)

$\tau_{q}=\frac{14.40516}{1.037\exp(0.09(V_{m}+30.61))+0.369\exp(-0.12(V_{m}+23.84))}+2.75352$ (37)

**Hyperpolarization-activated, “funny” current (*I*_f_)**

The channel current formulations are based on Wilders et al.[9] and Kurata et al. models.[6]

$I_{f}=I_{fNa}+I_{fK}$ (38)

$y_{\infty}=\frac{1}{1+\exp((V_{m}-V_{If,1/2})/13.5))}$ (39)

$\tau_{y}=\frac{0.7166529}{\exp(-(V_{m}+386.9)/45.302)+\exp((V_{m}-73.08)/19.231)}$ (40)

$I_{fNa}=C_{m}0.3833g_{If,\max}(V_{m}-E_{Na})y^{2}$ (41)

$E_{Na}=\frac{RT}{F}\ln(\frac{Na_{o}}{Na_{i}})$ (42)

$I_{fk}=C_{m}0.6167g_{If,\max}(V_{m}-E_{K})y^{2}$ (43)

**Sustained inward current (*I*_st_)**

The channel current formulations are based on Kurata et al. model.[6]

$I_{st}=C_{m}g_{st,\max}(V_{m}-E_{st})q_{a}q_{i}$ (44)

$q_{a,\infty}=\frac{1}{1+\exp(-(V_{m}+57)/5)}$ (45)

$\alpha_{qa}=\frac{1}{0.15\exp(-V_{m}/11)+0.2\exp(-V_{m}/700)}$ (46)

$\beta_{qa}=\frac{1}{16\exp(V_{m}/8)+15\exp(V_{m}/50)}$ (47)

$\tau_{qa}=\frac{1}{\alpha_{qa}+\beta_{qa}}$ (48)

$\alpha_{qi}=\frac{1}{3100\exp(V_{m}/13)+700\exp(V_{m}/70)}$ (49)

$\beta_{qi}=\frac{1}{95\exp(-V_{m}/10)+50\exp(-V_{m}/700)}+\frac{0.000229}{1+\exp(-V_{m}/5)}$ (50)

$\tau_{qi}=\frac{6.65}{\alpha_{qi}+\beta_{qi}}$ (51)

$q_{i,\infty}=\frac{\alpha_{qi}}{\alpha_{qi}+\beta_{qi}}$ (52)

**Na^+^-dependent background current (*I*_bNa_)**

$I_{b,Na}=C_{m}g_{bNa}(V_{m}-E_{Na})$ (53)

**Na^+^-K^+^ pump current (*I*_NaK_)**

The channel current formulations are based on Kurata et al. model.[6]

$I_{b,Na}=C_{m}I_{NaK,\max}\frac{1}{1+(K_{mKp}/K_{0})^{1.2}}\frac{1}{1+(K_{mNap}/Na_{i})^{1.3}}\frac{1}{1+\exp(-(V_{m}-E_{Na}+120)/30)}$ (54)

**Ca^2+^- background current (*I*_bCa_)**

$I_{b,Ca}=C_{m}g_{bCa}(V_{m}-E_{CaL})$ (55)

**Na^+^-Ca^2+^ exchanger current (*I*_NCX_)**

The channel current formulations are based on Dokos et al. model.[10]

$I_{NCX}=C_{m}k_{NCX}\frac{k_{21}x_{2}-k_{12}x_{1}}{x_{1}+x_{2}+x_{3}+x_{4}}$ (56)

$d_{o}=1+(Ca_{o}/K_{CO})\{1+\exp(Q_{CO}V_{m}/E_{T})\}+(Na_{o}/K_{1no})\{1+(Na_{o}/K_{2no})(1+Na_{o}/K_{3no})\}$(57)

$k_{43}=\frac{Na_{i}}{K_{3ni}+Na_{i}}$ (58)

$k_{41}=\exp[-Q_{n}V_{m}/(2E_{T})]$ (59)

$k_{34}=\frac{Na_{0}}{K_{3no}+Na_{0}}$ (60)

$k_{21}=\frac{(Ca_{0}/K_{co})\exp(Q_{CO}V_{m}/E_{T})}{d_{0}}$ (61)

$k_{23}=\frac{(Na_{0}/K_{1no})(Na_{0}/K_{2no})(1+Na_{0}/K_{3no})\exp(-Q_{n}V_{m}/(2E_{T}))}{d_{0}}$ (62)

$k_{32}=\exp[Q_{n}V_{m}/(2E_{T})]$ (63)

$x_{1}=k_{34}k_{41}(k_{23}+k_{21})+k_{21}k_{32}(k_{43}+k_{41})$ (64)

$\begin{matrix} d_{i}=1+(Ca_{sub}/K_{Ci})\{1+\exp(-Q_{Ci}V_{m}/E_{T})+Na_{i}/K_{cni}\}+ \\ (Na_{i}/K_{1ni})\{1+(Na_{i}/K_{2ni})(1+Na_{i}/K_{3ni})\} \end{matrix}$ (65)

$k_{12}=\frac{(Ca_{sub}/K_{ci})\exp(-Q_{Ci}V_{m}/E_{T})}{d_{i}}$ (66)

$k_{14}=\frac{Na_{i}}{K_{1ni}}\frac{Na_{i}}{K_{2ni}}(1+\frac{Na_{i}}{K_{3ni}})\frac{\exp(Q_{n}V_{m}/2E_{T})}{d_{i}}$ (67)

$x_{2}=k_{43}k_{32}(k_{14}+k_{12})+k_{41}k_{12}(k_{34}+k_{32})$ (68)

$x_{3}=k_{43}k_{14}(k_{23}+k_{21})+k_{12}k_{23}(k_{43}+k_{41})$ (69)

$x_{4}=k_{34}k_{23}(k_{14}+k_{12})+k_{21}k_{14}(k_{34}+k_{32})$ (70)

1. **Sarcolemmal membrane potential**

$\frac{dV_{m}}{dt}=-(I_{CaL}+I_{CaT}+I_{f}+I_{st}+I_{Kr}+I_{Ks}+I_{to}+I_{sus}+I_{NaK}+I_{NCX}+I_{b,Ca}+I_{b,Na})/C_{m}$ (71)

1. **Ca^2+^ buffering and diffusive transport between compartments**

**Ca^2+^ release flux (*j*_SRCarel_) from SR via RyRs**

The channel current formulations are based on Stern et al.[11] and were modified by Shannon et al.[12].

$j_{SRCarel}=k_{s}O(Ca_{jsr}-Ca_{sub})$ (72)

$k_{CaSR}=MaxSR-\frac{(MaxSR-MinSR)}{1+(EC_{50\_SR}/Ca_{jsr})^{HSR}}$ (73)

$k_{oSRCa}=k_{oCa}/k_{CaSR}$ (74)

$k_{iSRCa}=k_{iCa}/k_{CaSR}$ (75)

$\frac{dR}{dt}=(k_{im}RI-k_{iSRCa}Ca_{sub}R)-(k_{oSRCa}Ca_{sub}^{2}R-k_{om}O)$ (76)

$\frac{dO}{dt}=(k_{oSRCa}Ca_{sub}^{2}R-k_{om}O)-(k_{iSRCa}Ca_{sub}O-k_{im}I)$ (77)

$\frac{dI}{dt}=(k_{iSRCa}Ca_{sub}O-k_{im}I)-(k_{om}I-k_{oSRCa}Ca_{sub}^{2}RI)$ (78)

$\frac{dRI}{dt}=(k_{om}I-k_{oSRCa}Ca_{sub}^{2}RI)-(k_{im}RI-k_{iSRCa}Ca_{sub}R)$ (79)

**Ca^2+^ diffusion flux** **(*j*Ca_dif) from submembrane space to myoplasm**

$j_{Ca,dif}=(Ca_{sub}-Ca_{i})/\tau_{difCa}$ (80)

**The rate of Ca^2+^ uptake (pumping) (*j*_up_)** **by the SR**

The channel current formulations are based on Rudy et al.[13]

$j_{up}=P_{up}/(1+K_{up}/Ca_{i})$ (81)

**Ca^2+^ flux between (network and junctional) SR compartments (*j*_tr_)**

$j_{tr}=(Ca_{nSR}-Ca_{jSR})/\tau_{tr}$ (82)

**Natural Ca^2+^ buffering**

$\frac{df_{TC}}{dt}=k_{fTC}Ca_{i}(1-f_{TC})-k_{bTC}f_{TC}$ (83)

$\frac{df_{TMC}}{dt}=k_{fTMC}Ca_{i}(1-f_{TMC}-f_{TMM})-k_{bTMC}f_{TMC}$ (84)

$\frac{df_{TMM}}{dt}=k_{fTMM}Mg_{i}(1-f_{TMC}-f_{TMM})-k_{bTMM}f_{TMM}$ (85)

$\frac{df_{CMi}}{dt}=k_{fCM}Ca_{i}(1-f_{CMi})-k_{bCM}f_{CMi}$ (86)

$\frac{df_{CMs}}{dt}=k_{fCM}Ca_{sub}(1-f_{CMs})-k_{bCM}f_{CMs}$ (87)

$\frac{df_{CQ}}{dt}=k_{fCQ}Ca_{jSR}(1-f_{CQ})-k_{bCQ}f_{CQ}$ (88)

**Dynamics of Ca^2+^ concentrations in cell compartments**

$\begin{matrix} \frac{dCa_{i}}{dt}=\frac{(j_{Ca,dif}V_{sub}-j_{up}V_{nSR})}{V_{i}}-(CM_{tot}\frac{df_{CM_{i}}}{dt}+TC_{tot}\frac{df_{TC}}{dt}+TMC_{tot}\frac{df_{TMC}}{dt}) \\ \begin{matrix} \end{matrix}\begin{matrix} \end{matrix}\begin{matrix} \end{matrix}\begin{matrix} \end{matrix}\begin{matrix} \end{matrix}-\frac{\beta_{Ca}(j_{uni}-j_{NaCa,m})V_{myto}}{V_{i}} \end{matrix}$ (89)

$\begin{matrix} \frac{dCa_{sub}}{dt}=\frac{j_{SRCarel}V_{jSR}}{V_{sub}}-\frac{(I_{Ca_{L}}+I_{Ca_{T}}+I_{b,Ca}-2I_{NCX})}{2FV_{sub}}-(j_{Ca,diff}+CM_{tot}\frac{df_{CM_{s}}}{dt}) \\ \end{matrix}$ (90)

$\frac{dCa_{jSR}}{dt}=j_{tr}-j_{SRCarel}-CQ_{tot}\frac{df_{CQ}}{dt}$ (91)

$\frac{dCa_{nSR}}{dt}=j_{up}-j_{tr}\frac{V_{jSR}}{V_{nSR}}$ (92)

**References**

1. Maltsev VA, Lakatta EG (2010) A novel quantitative explanation for the autonomic modulation of cardiac pacemaker cell automaticity via a dynamic system of sarcolemmal and intracellular proteins. Am J Physiol Heart Circ Physiol 298: H2010-2023.

2. Nguyen MH, Dudycha SJ, Jafri MS (2007) Effect of Ca2+ on cardiac mitochondrial energy production is modulated by Na+ and H+ dynamics. Am J Physiol Cell Physiol 292: C2004-2020.

3. Cortassa S, Aon MA, O'Rourke B, Jacques R, Tseng HJ, et al. (2006) A computational model integrating electrophysiology, contraction, and mitochondrial bioenergetics in the ventricular myocyte. Biophys J 91: 1564-1589.

4. Nguyen MH, Jafri MS (2005) Mitochondrial calcium signaling and energy metabolism. Ann N Y Acad Sci 1047: 127-137.

5. Yaniv Y, Stanley WC, Saidel GM, Cabrera ME, Landesberg A (2008) The role of Ca2+ in coupling cardiac metabolism with regulation of contraction: in silico modeling. Ann N Y Acad Sci 1123: 69-78.

6. Kurata Y, Hisatome I, Imanishi S, Shibamoto T (2002) Dynamical description of sinoatrial node pacemaking: improved mathematical model for primary pacemaker cell. Am J Physiol Heart Circ Physiol 283: H2074-2101.

7. Demir SS, Clark JW, Murphey CR, Giles WR (1994) A mathematical model of a rabbit sinoatrial node cell. Am J Physiol 266: C832-852.

8. Zhang H, Holden AV, Kodama I, Honjo H, Lei M, et al. (2000) Mathematical models of action potentials in the periphery and center of the rabbit sinoatrial node. Am J Physiol Heart Circ Physiol 279: H397-421.

9. Wilders R, Jongsma HJ, van Ginneken AC (1991) Pacemaker activity of the rabbit sinoatrial node. A comparison of mathematical models. Biophys J 60: 1202-1216.

10. Dokos S, Celler B, Lovell N (1996) Ion currents underlying sinoatrial node pacemaker activity: a new single cell mathematical model. J Theor Biol 181: 245-272.

11. Stern MD, Song LS, Cheng H, Sham JS, Yang HT, et al. (1999) Local control models of cardiac excitation-contraction coupling. A possible role for allosteric interactions between ryanodine receptors. J Gen Physiol 113: 469-489.

12. Shannon TR, Wang F, Puglisi J, Weber C, Bers DM (2004) A mathematical treatment of integrated Ca dynamics within the ventricular myocyte. Biophys J 87: 3351-3371.

13. Luo CH, Rudy Y (1994) A dynamic model of the cardiac ventricular action potential. I. Simulations of ionic currents and concentration changes. Circ Res 74: 1071-1096.
